# Technoeconomic Insights into Metal Hydrides for Stationary Hydrogen Storage

**DOI:** 10.1002/advs.202415736

**Published:** 2025-04-03

**Authors:** Xinyi Wang, Peng Peng, Matthew D. Witman, Vitalie Stavila, Mark D. Allendorf, Hanna M. Breunig

**Affiliations:** ^1^ Lawrence Berkeley National Laboratory Berkeley CA 94720 USA; ^2^ Department of Mechanical Engineering California State University Fullerton Fullerton CA 92870 USA; ^3^ Sandia National Laboratories Livermore CA 94550 USA

**Keywords:** critical minerals, Hydrogen storage, metal hydride, techno‐economic analysis

## Abstract

Metal hydrides (MHs) are promising candidates for storing hydrogen at ambient conditions at high volumetric energy densities. Recent developments suggest hydride‐based systems can cycle and operate at favorable pressures and temperatures that work well with fuel cells used in stationary power applications. In this study, we present a comprehensive design and cost analysis of MH‐based long duration hydrogen storage facilities for a variety of power end users (0 to 20 megawatts (MW) supplied over 0 to 100 hours), to offer insights on technical targets for material development and operation strategies. Our findings indicate that hydride‐based storage systems hold significant size advantage in physical footprint, requiring up to 65% less land than 170‐bar compressed gas storage. Metal hydride systems can be cost competitive with 350‐bar compressed gas systems, with TiFe_0.85_Mn_0.05_ achieving $0.45/kWh and complex MH Mg(NH_2_)_2_‐2.1LiH‐0.1KH achieving $0.38/kWh. Extending charging times and increasing operating cycles significantly reduce levelized cost of storage, especially for complex MHs. Key strategies to further enhance the competitiveness of MHs include leveraging waste heat from fuel cells, reducing use of critical minerals, and achieving MH production costs of US$10/kg.

## Graphical abstract

Stationary hydrogen storage is essential for enabling the use of hydrogen and fuel cell technologies in applications such as backup power supply. This work evaluates the benefits of metal hydrides for storage, demonstrating the impacts of system design and operation, and material properties on viability. The analysis framework can be used to evaluate new materials and selection of practical applications.

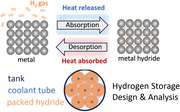

## 1 Introduction

Hydrogen is a promising solution for achieving a resilient power supply. It can be produced from a diversity of primary energy resources, while generating easily dissipated or recovered water and heat byproducts when used in fuel cells.^[^ ^1^ ^]^ Power outages occur in all countries, making reliable and low‐cost distributed long‐duration energy storage and power generation globally significant.^[^ ^2^ ^]^ Hydrogen‐powered fuel cells can replace traditional costly diesel backup generators which require deliveries of diesel, particularly when paired with on‐site production of hydrogen using locally available energy.^[^ ^3^ ^]^ Ongoing research efforts have focused on developing hydrogen fuel cell backup power systems in critical infrastructure applications.^[^ ^4^, ^5^, ^6^ ^]^ However, the infrastructure enabling long‐duration storage of bulk hydrogen has been highlighted as a concerning source of cost, energy and land requirements and a potential source of leakage.^[^ ^7^, ^8^ ^]^ Solid state hydrogen storage using metal hydrides (MHs) presents a promising alternative as these materials can store hydrogen at ambient conditions with high volumetric energy density, mitigating many of the inefficiencies of cryogenic or compressed gas storage.^[^ ^9^, ^10^, ^11^ ^]^

Two main categories of MHs for reversible hydrogen storage including intermetallic and complex MHs.^[^ ^1^ ^]^ Intermetallic hydrides are formed by combining hydrogen atoms with intermetallic compounds composed of two or more metals. The principle behind intermetallic hydrides is that an alloy A_x_B_y_H_z_, containing one element (A) that binds hydrogen strongly and another (B) that binds hydrogen weakly, can exhibit intermediate hydrogen storage properties between those of its constituent elements.^[^ ^12^, ^13^ ^]^ In practice, a limited number of intermetallic hydride structures, such as those with AB, AB_2_ or AB_5_ crystal structures, are commonly proposed for hydrogen storage applications.^[^ ^14^ ^]^ Complex MHs store hydrogen in a complex anion form bonded to a metallic cation, offering high hydrogen storage capacity.^[^ ^12^ ^]^ Despite their high volumetric energy density, recent system‐level performance of MH‐based hydrogen storage have primarily focused on transportation applications.^[^ ^15^, ^16^, ^17^, ^18^, ^19^, ^20^, ^21^ ^]^ Although there has been recent progress in developing these materials for stationary long‐duration energy storage applications, there is a notable lack of benchmarking of key material‐level and system‐level performance targets against incumbent systems, such as compressed gas storage. Furthermore, a comprehensive screening and evaluation approach, along with system design and balance‐of‐plant strategies to identify critical performance indicators, has yet to be developed.

In this study, we present novel process designs for metal hydride‐based hydrogen storage systems used in backup power applications and suggest targets for materials development. Our base case simulates a 10 megawatt (MW) 96‐h scale backup power system, representing the backup power and energy requirements for critical infrastructure such as hospitals or data centers.^[^ ^22^ ^]^ We adapt and employ a techno‐economic analysis (TEA) approach for material screening and system evaluation developed previously for metal organic framework (MOF) storage materials^[^ ^23^ ^]^ in order to benchmark promising candidate hydrides with adequate experimental absorption/desorption data. From this data we calculate hydrogen uptake under various pressure and temperature conditions in the tank and determine the tank size and balance of plant designs to supply necessary heating, compression, and cooling. The results are benchmarked against 170‐ and 350‐bar compressed gas storage operating under the same end user cycling scenarios. Finally, we consider mineral consumption implications and discuss research gaps and offer a roadmap for material and system research that can further improve viability of this critical infrastructure advancement.

## 2 Experimental Section

### 2.1 Back‐Up Power System and Operation Cycles

**Figure 1:**
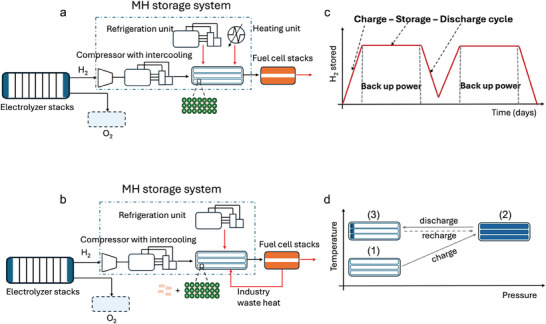
a) Process flow for the base case scenario of a MH‐based hydrogen storage back‐up energy system, with main unit operations and system boundaries indicated by the dashed box. b) Process flow for the alternative scenario featuring both gas‐phase hydrogen and metal hydride hydrogen storage; here, gas phase hydrogen initiates the fuel cell operation, which in turn provides the waste heat required for the hydrogen desorption from the metal hydride. c) Schematic diagram illustrating the system cycles for charging, storing, discharging, and recharging. d) Overview of the temperature and pressure conditions across various stages.

In the base case, metal hydride hydrogen storage systems coupled with co‐located electrolyzers and fuel cells sized is modeled to meet 10 MW backup power events lasting 96 h.^[^ ^22^ ^]^ In other words, the system can supply 10 MW power for 96 h without recharging. The power scale, discharge duration, hydrogen release efficiency and fuel cell efficiency determine the total hydrogen storage requirement, with larger power scales or longer discharge durations necessitating greater hydrogen storage capacity. For the base case, charging time is set to 96 h. the effects of varying charging rates, including both fast and slow charging scenarios are also analyzed, which will be discussed later. **Figure** **1**a illustrates the base case process flow of a metal hydride‐based hydrogen storage backup energy system. In this on‐site stationary hydrogen backup system, hydrogen is generated by alkaline water electrolyzer stacks and stored in metal hydride‐packed storage tanks. The storage system also includes ancillary facilities to manage cooling, compression, and heating requirements during the charge and discharge cycles. Hydrogen is reconverted to electricity via proton‐exchange membrane fuel cell stacks when needed.

The operational cycle of the storage system is shown in Figure 1d. Initially, the storage tanks are set to be at room temperature. During the initial charging stage, the tanks are heated to the required operating temperature, and hydrogen from the electrolyzer is compressed and passed into the tanks to reach stage 2). During the absorption process, hydrogen reacts with material to form hydrides, releasing heat. To maintain operating conditions and ensure efficient absorption, the tanks are actively cooled using coolants circulate through cooling tubes. After hydrogen absorption, the tanks are sealed, allowing the system to cool to room temperature, thereby storing hydrogen for extended periods. This design decision is based upon a cost comparison of ambient storage with reheating versus warm storage with thermal insulation. The costs of these two approaches are calculated: the first one is a stand‐by mode, where after the charging, the system is allowed to cool to room temperature. When hydrogen needs to be discharged, the tank is reheated to the required operating temperature. The second approach involves maintaining the tank at a consistent temperature between the charging and discharging processes through continuous heating and insulation. The cost for these two methods is shown in Figure S6 (Supporting Information). As the number of annual charging and discharging cycles increases, the time between these processes shortens, reducing the energy cost required to maintain a constant temperature. However, the cost for the standby mode increases due to the need for more frequent reheating. Despite this, the standby mode still proves to be less expensive over the full cycle span, which is why it is selected for further analysis.

When hydrogen is needed for power generation, the storage system is reheated to the same temperature, releasing hydrogen from the tanks to the fuel cell stacks at 2 bar, reaching stage 3). Some hydrogen remains in the tank due to the release efficiency of the hydride materials. During this release, heat is supplied to maintain the temperature necessary for hydrogen desorption (∆H_des_). After each discharge process, hydrogen for the next cycle is immediately recharged to full storage capacity (stage (2)), as shown in Figure 1c. In the base case scenario, 12 charge and discharge cycles per year is set, corresponding to 1152 h of annual power outage. The impact of the number of cycles is investigated and discussed in the results. A higher number of cycles implies more frequent use of the storage system, which naturally affects the system's levelized cost of storage (LCOS).

Additionally, a process flow is developed for an alternative scenario as shown in Figure 1b where there is more gas‐phase hydrogen in the storage tank. For certain intermetallic hydrides, if the operating temperature remains below 80 °C, the heat from the fuel cell coolant is sufficient to facilitate hydrogen desorption and reduce operational costs.^[^ ^24^ ^]^ In this setup, the storage tanks are oversized to allow for 5 wt.% gas phase hydrogen alongside hydrides in the storage tanks. The gas phase hydrogen is released first to activate the fuel cells, whose waste heat, conveyed by a coolant fluid, is then utilized to heat the metal hydride storage system for hydrogen desorption.

### 2.2 Material Selection and Storage Tank Models

We select representative MHs and take experimental pressure‐composition‐temperature (PCT) curves from the literature for AB (TiFe and TiFe_0.85_Mn_0.05_),^[^ ^25^ ^]^ AB2 (Ti_0.95_Zr_0.05_Mn_1.55_V_0.45_Fe_0.09_),^[^ ^26^ ^]^ AB5 (MnNi_4.6_Fe_0.4_)^[^ ^26^ ^]^ intermetallic hydrides and complex MH (Mg(NH_2_)_2_‐2.1LiH‐0.1KH).^[^ ^18^, ^27^ ^]^ Under typical hydrogen storage pressure/temperature swing constraints, intermetallic hydrides generally have usable capacities less than 2 wt.% hydrogen,^[^ ^12^, ^14^ ^]^ although some high entropy alloys exhibit saturation capacities exceeding 3% and hydrogen‐to‐metal ratios (H/M) exceeding 2.^[^ ^28^, ^29^ ^]^ Common advantages of intermetallic MHs are rapid and reversible hydrogen charging and discharging rates, high volumetric densities, and their operation at near‐ambient temperatures and low pressures.^[^ ^30^ ^]^ Complex hydrides exhibit higher hydrogen gravimetric uptakes, typically ranging from 4 to 14 wt.% hydrogen.^[^ ^31^ ^]^ However, they are limited by slow kinetics, requiring higher operating temperatures and pressures for effective use.^[^ ^1^, ^30^, ^32^ ^]^ the usable hydrogen uptake is determined from experimentally measured PCT curves, within a specific operating pressure range and temperature, as shown in Figure S1 (Supporting Information). A 2‐bar pressure limit during the desorption is chosen to facilitate hydrogen flow from the storage tanks to the fuel cell stacks. The material properties are also obtained such as density, heat capacity, thermal conductivity and absorption enthalpy from literature (Table S2, Supporting Information).

For the base case scenario, each storage tank includes multiple metal hydride units with internal cooling and heating tubes, as shown in Figure S2a (Supporting Information). During the hydrogen charging process, the metal hydride generates heat, which must be effectively removed to sustain continuous hydrogen uptake. Conversely, during the discharging process, sufficient heat must be supplied to maintain the necessary temperature. Coolant or heating steam is circulated through these tubes to facilitate efficient heat transfer, thereby enhancing the charging and discharging kinetics. The number and spacing of the cooling tubes within the tank are calculated using the adiabatic form of the metal hydride acceptability envelope in cylindrical coordinates, a model previously developed by Corgnale et al.^[^ ^33^ ^]^ Based on the number of tubes, the internal diameter of the tank, and outer dimensions of the storage tank can be estimated using the “Tankinator” model and “Design Tool” developed at Pacific Northwest National Laboratories.^[^ ^21^, ^34^, ^35^ ^]^ To fine‐tune the parameters, all codes are integrated into Python, rather than using the previously developed Excel Visual Basic for Application code. To provide a comparison with the base case tank design, an alternative tank configuration is also evaluated as depicted in Figure S2b (Supporting Information). In this design, an external cooling tube surrounds the storage tank is used. However, the limited contact surfaces require a smaller tank diameter to ensure adequate heat transfer between the tank and the coolant. Effects of these two tank designs on the system performance are investigated and included in Note S1 and Figure S2c,d (Supporting Information).

Key system‐level assumptions in this analysis include first, the temperature dependence of the heat capacity of MH and storage tanks is neglected. Second, detailed internal mass and heat transfer effects within the storage tanks are not considered, including complex interactions between hydrogen gas and the storage medium, such as hydrogen diffusion and leakage. The thermal conductivity and heat capacity of MHs and storage tanks are set to remain constant, regardless of hydrogen concentration or temperature. Third, the complex kinetics of MHs are acknowledged but not included in the TEA analysis. Lastly, heat exchange and insulation between the ambient and the storage system were found to have negligible cost and therefore were not included in the final TEA analysis.

### 2.3 System‐Level Performance and TEA

Based on the system boundary, operational cycles, material selection and tank design described above, an analysis of the system‐level performance of the MH hydrogen storage system, focusing on volumetric energy density, footprint, and cost performance is conducted. The volumetric energy density is calculated by dividing the energy released from each storage tank by the tank's total volume. Based on the size of each tank, the total number of tanks, the vertical stacking arrangement, the separation distance between tanks and the overall safety distance for the storage system (Figure S7, Supporting Information), the required footprint is estimated for the entire storage system. Then analyze the LCOS of the MH hydrogen storage system by normalizing the capital investment and operational costs over the system's operating lifetime against the output power. The power and energy required to size and compute the capital and operation costs of various equipment are determined using an energy balance method across the different operation stages.^[^ ^23^, ^36^ ^]^ Additional costs, such as maintenance, labor, land price, and direct and indirect cost, are also considered. More detailed cost factors are provided in Table S5 (Supporting Information) with detailed TEA modeling available in Note S2 (Supporting Information). The effects of storage tank size on LCOS, various methods of determining labor costs, and insulation considerations are presented in Figures S3–S5 (Supporting Information), respectively. In addition to our base case scenario, it also investigates the effects of charging rate, number of cycles, and power scales on LCOS. The performance of MH‐based hydrogen storage systems is compared to conventional compressed gas hydrogen storage at 170 and 350 bar. In contrast, onboard transportation applications use 700 bar pressure to maximize storage capacity, which requires expensive high‐pressure storage tanks that have yet to be demonstrated for large storage capacities.^[^ ^37^ ^]^ Therefore, such high‐pressure storage is not considered for large stationary applications and is not analyzed in this study.

## 3 Results and Discussion

### 3.1 Performance of MH‐Based Hydrogen Storage

**Figure 2:**
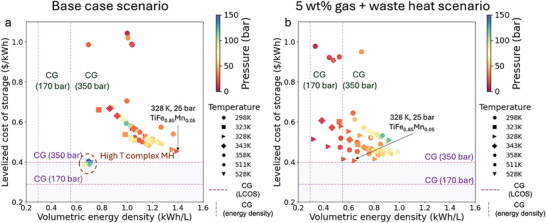
Performance comparison between MHs and compressed gas hydrogen storage methods for a 10 MW system with 96 h of discharge capacity. a) Base case scenario: LCOS and volumetric energy density of MHs across various operating temperatures and pressures, benchmarked against compressed gas storage. b) Alternative scenario: LCOS and volumetric energy density for intermetallic MHs storing 5 wt.% of hydrogen in the gas phase to initialize the fuel cell, with waste heat from fuel cells utilized to drive the discharge process. All scenarios assume 12 charge‐discharge cycles per year.

**Figure** **2** presents the system‐level volumetric energy density and LCOS for selected MHs compared to compressed gas hydrogen storage. The LCOS of the storage system represents the annualized equivalent of fixed capital investment and operational costs – including energy, labor maintenance and other costs related to the stored hydrogen per year, excluding the electrolysis and fuel cells. Figure 2a illustrates the performance of MHs under various operating temperatures and pressures (denoted by different symbols and colors, respectively) in the base case scenario. All MHs demonstrate a higher volumetric energy density than compressed gas, ranging from 1.2 to 2.5 times greater than 350 bar compressed gas, and 2.24 to 4.74 times greater than 170 bar compressed gas storage system. This clearly shows the advantage of requiring smaller land footprint. For the LCOS, all intermetallic MHs exhibit a higher LCOS (ranging from $0.48/kWh to $1.27/kWh depending on the operating conditions) compared to compressed gas systems. Among the intermetallic MHs, TiFe_0.85_Mn_0.05_ at 328 K and 25 bar has the lowest cost ($0.45/kWh), attributed to its combination of relatively high gravimetric capacity (1.84%), low absorption pressure (25 bar), moderate operating temperature (328 K) and mild absorption enthalpy (32.5 kJ mol^−1^).^[^ ^25^ ^]^ In contrast, the complex MH system (Mg(NH_2_)_2_‐2.1LiH‐0.1KH), under its optimal operating conditions, has a slightly lower LCOS ($0.38 per kWh) than 350 bar compressed gas ($0.40 per kWh), primarily due to their higher hydrogen uptake, which reduces the amount of MH and consequently lowers the overall material cost. The cost of MHs is a major component of the system cost, and a reduction in material cost offsets the increased cost associated with more demanding operating conditions (higher temperature, higher pressure and larger heat management needs) for complex MH system. It is important to note that the system cost performance is highly sensitive to the actual cost of MHs, which is set at $20/kg for all MHs in the base case scenario.

Figure 2b shows the results for the second scenario, where each tank stores an additional 5 wt.% gas‐phase hydrogen to initiate fuel cells, and the fuel cells' waste heat supplies the required desorption heat for the intermetallic MHs. The storage costs are reduced, narrowing the gap with the LCOS of 350 bar compressed gas and enhancing economic competitiveness of MHs. However, this approach comes with a tradeoff: storing more gas‐phase hydrogen reduces the system's volumetric energy density. Among the intermetallic MHs, TiFe_0.85_Mn_0.05_ at 328 K and 25 bar achieves the lowest LCOS ($0.41/kWh) while maintaining a good balance with a volumetric energy density of 0.65 kWh L^−1^.

**Figure 3:**
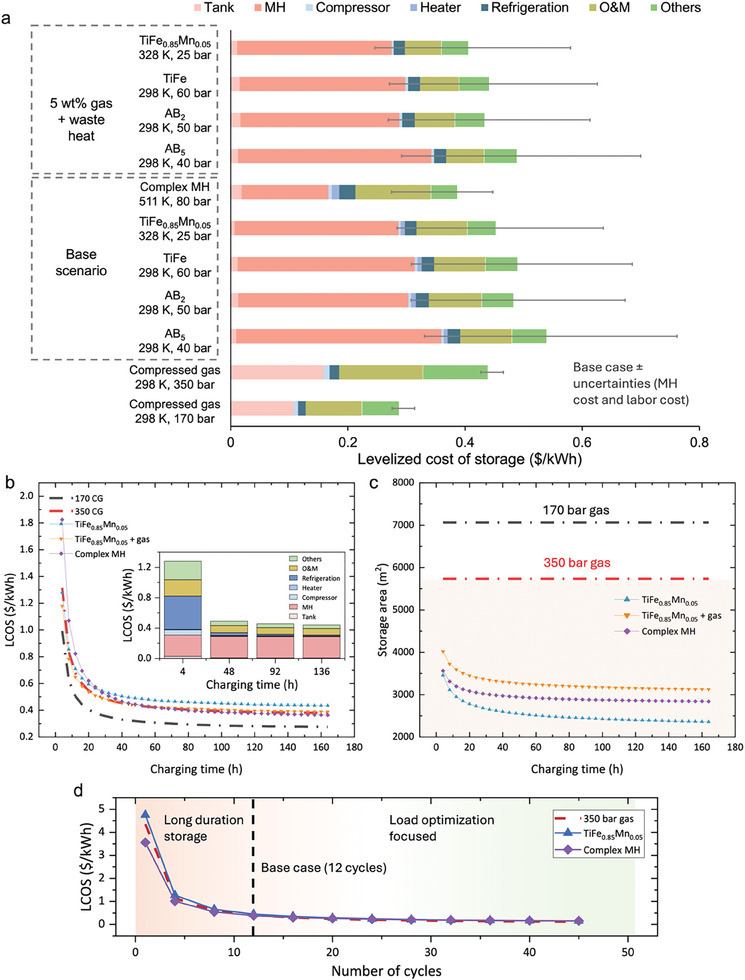
Cost breakdown of MHs and comparison with compressed gas hydrogen storage methods for a 10 MW system with 96 h of discharge capacity. a). LCOS breakdown for selected MHs hydrogen storage in two scenarios compared with compressed gas hydrogen storage. “Others” represents the direct and indirect costs such as installation cost, piping, contingency, legal and insurance, and site preparation. “O&M” refers to operation and maintenance. Error bar accounts for variations in labor cost ($25–$60 per h) and MH cost ($10–$30 per kg). b) The effects of charging time on the LCOS for MHs and compressed gas. The inset graph shows the effects of charging time on the LCOS breakdown for the TiFe_0.85_Mn_0.05_ base case scenario. c) Effects of charging time on the required storage space for MHs and compressed gas. d) Effect of annual operating cycles on the LCOS for representative MHs and 350‐bar compressed hydrogen gas. The color gradient in the background highlights different application focuses: orange represents long‐duration, backup‐focused applications, while blue represents load optimization‐focused applications requiring short‐term storage between charging and discharging. The black dashed vertical line indicates 12 annual cycles, corresponding to the base case scenario.

**Figure** **3**a provides the LCOS cost breakdown for MHs and compressed gas systems. The cost of MHs is the main component of the capital cost for MH‐based hydrogen storage, whereas 350 bar compressed gas incurs higher storage tank costs due to the use of Type 3 tanks. Mg(NH_2_)_2_‐2.1LiH‐0.1KH requires lower investment in the storage material due to its third times higher hydrogen uptake compared to intermetallic MHs. The error bars represent variations in labor costs ($25 to $60 per h) and MH costs ($10–$30 per kg). At a material cost of ≈$15 per kg, the LCOS of MH systems is comparable to that of 350‐bar compressed gas. If MH costs are further reduced to $10 per kg, the LCOS becomes lower than 350‐bar compressed gas, making MH systems economically favorable. In the 5 wt.% gas and waste heat scenario, similar trends are observed as in the base case, with a marginal impact on overall costs due to the inclusion of external heat, which reduces the capital and operational costs of the heater. Since the capital investments in equipment are related to power capacity, their costs become more economical when slower charging (smaller power rating) is allowed, as presented in Figure 3b. This effect is more pronounced in complex MH case (purple line in Figure 3b), where cost for heat management constitutes a large portion of system costs. The required storage space for MHs decreases notably with longer charging times (Figure 3c). This is because the required number of coolant tubes inside each tank decreases with slower charging, leading to a smaller tank size and an overall reduction in storage area. MH systems show a much smaller required storage area compared to the compressed gas system due to their high volumetric energy density. While onshore plants may have sufficient land for large storage facilities, the price of materials becomes an important factor; in contrast, for offshore applications or backup power in high‐land‐cost urban areas, space is constrained, making compact storage more desirable.^[^ ^38^ ^]^ Additionally, the complex MH require fewer storage tanks to deliver the same power compared to compressed gas, reducing installation, maintenance, and infrastructure costs.

The effects of annual operating cycles on LCOS are shown in Figure 3d. Increasing the number of cycles reduces the capital cost per cycle (or per energy produced), as the system is utilized more frequently, leading to a decrease in the marginal cost of capital—an effect that enhances economic efficiency. With fewer cycles, the storage time between charging and discharging is extended; for instance, one cycle per year implies storing hydrogen for 357 days after charging, which characterizes long duration energy storage. In contrast, a higher number of cycles means that hydrogen is stored for a shorter period before being discharged for energy generation. Compared to 350‐bar compressed hydrogen gas, intermetallic TiFe_0.85_Mn_0.05_ has a higher capital cost, resulting in a higher LCOS at low cycle frequencies. However, as the number of cycles increases, the economies of scale come into play, leading to a sharper decrease in LCOS for TiFe_0.85_Mn_0.05_, narrowing the difference with 350‐bar gas. Mg(NH_2_)_2_‐2.1LiH‐0.1KH, on the other hand, have lower capital costs due to the reduced amount of MH required (Figure S8, Supporting Information), but they incur higher operational energy costs. While the lower capital costs keep the LCOS for complex MH low at a small number of cycles, the impact of capital cost savings diminishes as the number of cycles increases. In contrast, the higher operational costs do not benefit from increased cycling. As a result, with further cycling, the operational costs eventually outweigh the capital savings, potentially causing the LCOS for Mg(NH_2_)_2_‐2.1LiH‐0.1KH to surpass that of the other two methods. In summary, intermetallic MHs with higher capital costs due to the larger amount of MH used are more favorable when the system is used frequently. Meanwhile, complex MH Mg(NH_2_)_2_‐2.1LiH‐0.1KH with lower MH costs are more competitive when the system is used less frequently.

### 3.2 Effects of Material Properties and Future Development Targets

**Figure 4:**
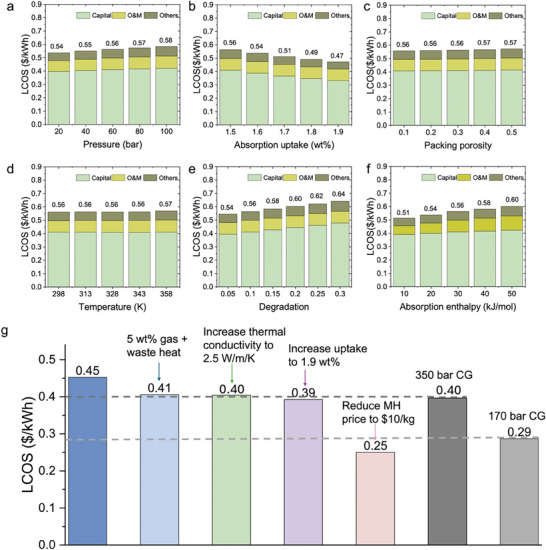
Effects of material properties on LCOS and material targets. a–f) The panels illustrate the effects of varying individual material properties on the LCOS and the corresponding cost breakdown. Each panel examines the impact of changing one specific property at a time. g) A roadmap for reducing LCOS of an H_2_ storage system, starting from the base case of TiFe_0.85_Mn_0.05_ under 328 K and 25 bar. The diagram highlights the reduction in LCOS achievable through various methods and compares these results with compressed gas storage systems.

To better understand the effects of individual material properties on the overall LCOS, we conduct a sensitivity analysis using a hypothetical MH system. We set this MH has a hydrogen uptake of 1.5 wt.% at 333 K and 60 bar pressure, and a release efficiency of 90% (resulting in a usable hydrogen release of 1.35 wt.%). The enthalpy of hydrogen absorption is assumed to be 30 kJ mol^−1^. By varying each material property independently, while holding the others constant, we calculate the resulting LCOS, as shown in **Figure** **4**a–f. For instance, when the operating pressure is varied from 20 bar to 100 bar, at constant uptake and power, the LCOS increases from 0.54 $/kWh to 0.58 $/kWh (Figure 4a). This increase is primarily due to the higher costs associated with the storage tank and the expenses of the compressor. Conversely, an increase in hydrogen uptake decreases the LCOS, as it reduces the required amount of MH (Figure 4b), thereby decreasing costs associated with both the storage tank and related equipment. Packing porosity refers to the voids between the MH particles when they are packed together. As shown in Figure 4c, packing porosity has minimal impact on LCOS, as it slightly increases storage tank costs, which represent only a small fraction of the overall system cost. However, it is important to note that loose packed MHs affect volumetric energy density and increase the required land footprint. They remain feasible when space constraints are not critical, as they provide additional room for MH expansion during hydrogen absorption. Additionally, material degradation also plays a critical role in LCOS. Over the system's lifetime, storage capacity decreases due to MH degradation. Here, we account for this degradation rate by adjusting the upfront required MH material purchase by 5% to 30%. For instance, a 0.05 (5%) degradation rate means that 5% more MHs must be purchased to compensate for the eventual reduction in hydrogen storage capacity. As shown in Figure 4e, improving MH cyclability and minimizing degradation significantly enhance economic feasibility as well as reduces dependency on critical minerals.

While the above analysis isolates each parameter, it should be noted that many material properties are correlated. For example, from the van't Hoff equation, the absorption enthalpy is a function of the equilibrium pressure and operating temperature, and materials with higher uptake typically exhibit higher absorption enthalpy due to stronger metal‐hydrogen bonding interactions.^[^ ^39^ ^]^ Our analysis highlights the significance of each material property on the LCOS. For example, a 0.4 wt.% increase in hydrogen uptake can reduce the LCOS by 0.09$ per kWh. Even if achieving this uptake requires higher operating temperatures or enthalpy, the overall cost reduction may still justify the trade‐off, as savings from increased uptake outweigh the additional costs. However, a more detailed analysis of the relationship between uptake and enthalpy is needed in future work, as understanding the rate at which enthalpy changes with uptake is critical for determining cost trade‐offs.

Figure 4g presents the potential for reducing the LCOS for the current MHs. The roadmap details the storage system costs using TiFe_0.85_Mn_0.05_ under conditions of 328 K and 25 bar as the base case. In this scenario, the cost is ≈1.2 times higher than that of 350 bar compressed gas, and 1.6 times higher than 170 bar compressed gas. Cost reduction can be achieved through advancements in material properties and engineering design, such as leveraging waste heat for enhanced thermal management, improving material thermal conductivity, increasing hydrogen uptake and reducing the cost of MHs. In the near term, known improvements in material manufacturing and the use of low‐cost raw material are expected to reduce MH costs. As noted in Figure 4g, by decreasing the MH price to $10 per kg, MH‐based hydrogen storage could outperform 170 bar compressed gas in LCOS.

### 3.3 System Scalability

**Figure 5:**
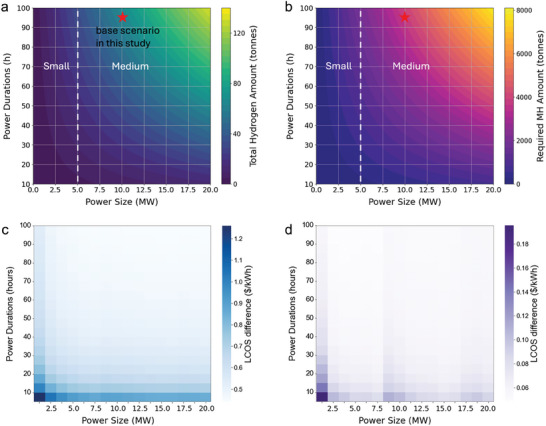
Scalability of the hydrogen storage system. a,b) Total stored hydrogen and required MHs amounts as functions of power durations and power size, respectively. c) LCOS for TiFe_0.85_Mn_0.05_ at 328 K and 25 bar across various power scales. d) LCOS difference between TiFe_0.85_Mn_0.05_ at 328 K and 25 bar and a 350‐bar compressed gas system.

While solid state hydrogen technologies can be modular, providing exceptional scalability for energy storage in a range of power applications, there are important scaling factors worth considering. **Figure** **5**a,b shows the total mass of stored hydrogen and MH material corresponding to power requirements. The power duration refers to the time that the system can continuously supply energy; in our base case, this is set at 96 h. As power duration and size increases, more hydrogen needs to be stored, thereby requiring a larger amount of MH. For example, supplying 20 MW for 100 h would require nearly 8000 tonnes of intermetallic TiFe_0.85_Mn_0.05_ MH, while the complex MH Mg(NH_2_)_2_‐2.1LiH‐0.1KH, with its higher hydrogen uptake, reduces the required amount to ≈3000 tonnes. While it might be possible to limit MH applications to those that require minimal amount of material, we show in Figure 5c,d that MH tend to outcompete compressed gas systems only at larger sizes, above 3.75 MW in medium to long durations storage applications. A system with a power size of 5 MW or below is sufficient for supporting a small data center, whereas a power size between 5 and 20 MW would be suitable for a medium‐sized data center.

With increasing power size or duration, more MH material is required, as shown in Figure 5b. This raises concerns about the feasibility of large‐scale material production. The typical synthesis method for MHs is ball milling. While this process is feasible at the lab scale, it presents challenges when scaled to industrial levels. For example, during the ball milling of a large‐scale 6 kg TiFeMn sample, a significant portion of the material adhered to the inner surface the milling drum and the milling balls. As a result, only ≈2 kg of TiFeMn was recovered after milling.^[^ ^40^ ^]^ Material loss increases both the raw material requirements and production costs, and we expect future work will target such inefficiencies. Additionally, the TiFeMn alloys produced at an industrial scales can exhibit different phase fractions and microstructures, which affect hydrogen sorption properties.^[^ ^41^ ^]^ This variation is primarily due to oxygen contamination introduced during synthesis, either from less pure raw materials or from exposure to air during processing, especially when vacuum conditions are suboptimal. Limiting oxygen content requires the use of high‐purity raw materials and stringent processing conditions, which can further raise production costs. As deployment must scale beyond an individual facility to make a broader impact, MH‐based storage systems could face supply risks due to the nascency of manufacturing methods and the dependency on critical materials, discussed in the next section.^[^ ^42^, ^43^ ^]^

### 3.4 Material Criticality

**Figure 6:**
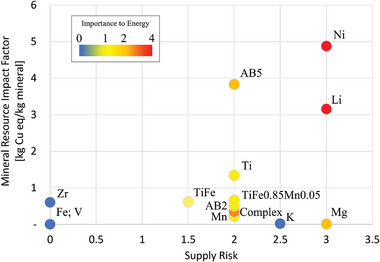
Supply risk and mineral resource impact factor for individual elements and metal hydrides (MHs) evaluated in this study. Each data point is color‐coded based on the material's importance in energy‐related applications.

Herein, we evaluate material criticality of MH by focusing on three key factors: supply risk, a life cycle mineral resource impact factor, and the importance of the material in energy‐related applications. Supply risk is influenced by several factors, including geopolitical stability, the concentration of material production in specific regions, and the limited availability of the raw materials.^[^ ^44^ ^]^ High supply risks can threaten the reliability and cost‐effectiveness of MH technologies, particularly when dependence on a few countries or volatile regions affects material availability. The importance of the material in energy‐related applications refers to how crucial the material is for current or emerging energy technologies, such as batteries, fuel cells, and renewable energy systems. These two factors are both considered by the United States and European Union when defining minerals as critical, however there are no clear thresholds limiting technology development. As such, we use iridium in PEM electrolyzers as a benchmark, where iridium use has been projected to reach 20% of global annual iridium production in the year 2023. In contract, the life cycle mineral resource impact factor is a quantitative metric, measured in kg Cu equivalents per kg of mineral consumed. Considered a “mid‐point” impact, mineral resource is ultimately an indicator on the cost of future extraction and depletion of mineral resources. **Figure** **6** presents the scores for MH included in this study based on chemical composition, not including potential manufacturing losses. Each data point corresponds to either individual elements or MH assessed in this study, color‐coded based on their criticality in relation to energy applications.

Minerals such as Ni and Li exhibit both high material criticality and significant mineral resource impacts. This combination underscores substantial risks concerning supply security and environmental impact. These risks extend to their corresponding hydrides, such as AB_5_ (MnNi_4.6_Fe_0.4_) and complex MH (Mg(NH_2_)_2_‐2.1LiH‐0.1KH), where we estimate only 250 and 420, 10 MW power facilities could be built before exceeding 20% global annual production of a mineral, respectively. Magnesium demonstrates a low mineral resource impact factor but poses a supply risk similar to that of Ni and Li. This is noteworthy since Mg contributes significantly to complex MH formulations, where its supply dynamics could influence overall material availability. Titanium shows moderate risks in both material criticality and environmental impact. These risks are further mitigated when Ti is alloyed with Fe, which has a very low criticality in all assessed categories, allowing for ≈1200 10 MW power facilities to be constructed without exceeding 20% global annual production of a mineral. Notably, while not considered critical, V is produced as a byproduct and limits AB_2_ scaleup to only 42 power facilities unless new sources are developed. A comprehensive understanding of the interplay between material criticality and environmental impact is essential for designing our future energy storage solutions. As such, we provide estimates of global warming potential related to MH synthesis in Note S8 (Supporting Information). We estimate these potentials, which can be thought of as the embodied emissions of the MH, are similar to those of Mg, and Ni, and are notably half the impact of Ti and a quarter of the impact of Li.

### 3.5 Uncertainties and Future Direction

In this study, we conduct a system‐level cost analysis of MH‐based hydrogen storage for backup power applications and benchmark it against compressed gas method. Our results suggest that MHs offer a compelling combination of high volumetric energy density and low operating pressures compared to compressed gas hydrogen storage. These advantages not only reduce the required footprint but also reduce safety concerns and system complexity. Intermetallic MHs could potentially achieve cost parity with 350 compressed gas in terms of LCOS when integrated with external heat sources, such as waste heat from fuel cells. Additionally, allowing for a slower charge rate could further decrease the LCOS and reduce the system footprint for MHs. Among all the expenditures, the material cost of MHs is the most significant capital expense in the storage system. Therefore, future advancements in MH technology – such as developing MHs with higher uptakes or using less expensive raw materials and enhanced manufacturing techniques‐ could further reduce costs, making MHs even more competitive. Beyond evaluating the overall cost performance of this emerging technology, our study also identifies key limitations and critical areas for future research, as outlined below.

Our analysis is grounded in experimental measurements of pressure composition temperature (PCT) curves for hydrogen absorption and desorption in MHs. Accurate measurements of hydrogen uptake and corresponding PCT curves are important for assessing hydrogen capacity and heat enthalpy. However, variations in hydrogen uptake variations can occur even with identical compositions due to factors such as synthesis variations, impurities, phase separation and insufficient activation. Furthermore, scaling up hydrogen storage introduces additional variations, as MHs synthesized on an industrial scale may exhibit different hydrogen absorption properties compared to those synthesized in the laboratory.^[^ ^41^ ^]^

For long‐duration hydrogen storage, which requires large amount of MH, standardizing industrial‐scale material synthesis and accurately quantifying hydrogen absorption performance are critical areas for future research. In addition to experimental data, accurate simulation results can further explore the potential of MHs in hydrogen storage. Unlike the extensive molecular simulations and machine learning investigations of hydrogen uptake in physisorption‐based MOFs,^45–[^ ^48^ ^]^ the complexity of hydrogen absorption by MH, encompassing physisorption, chemisorption, diffusion, and phase transformations, is computationally expensive to simulate. Although machine learning (ML) has been used to predict hydrogen diffusion in MHs,^[^ ^49^ ^]^ hydride formation enthalpy,^[^ ^50^ ^]^ thermodynamic stability^[^ ^51^ ^]^ and phase diagrams^[^ ^52^ ^]^ future research should target using standardized experimental methods or computational simulations to generate sufficient data for MH‐based hydrogen storage. These data could then be employed to interpolate hydrogen absorption performance using ML models.

The primary capital cost for MH‐based hydrogen storage is attributed to the price of MHs. In this study, we use a uniform price of $20 per kg for all MHs tested, with error margin ranging from $10–$30 per kg. However, this is a rough estimate, as MHs generally have varying prices. For example, AB and AB_2_‐type hydrides can be cheaper than AB_5_ hydrides due to AB_5_ containing higher‐cost rare‐earth elements. The price of MHs comprises two major components: raw material costs and manufacturing costs. The raw material cost depends on the elements used; For instance, Mn, Al, Fe, Mg, Cr, and Ti are relatively inexpensive, while Zr, Mo, W, Co, and V are more costly.^[^ ^53^, ^54^ ^]^ The manufacturing cost of MHs, which is highly dependent on synthesis methods and production scale, remains underexplored.^[^ ^39^ ^]^ A more detailed analysis of MH prices is crucial for future research to provide a more accurate commercial potential assessment for MHs in hydrogen storage.

Metal hydride reactions require efficient heat removal during the charging and heat supply during discharging. Heat management poses challenges due to the poor heat transfer characteristics of MH powders, which can potentially reduce the reaction rate. To mitigate this limitation, we use a tank designed to enhance heat transfer. In each tank, we calculate the required number of coolant tubes to achieve the desired charging and discharging rate. Additionally, compaction of powders into pellets or plates, supplemented with expanded natural graphite (ENG), is a common method to improve thermal conductivity and packing density^[^ ^55^ ^]^ As shown in Note S4 (Supporting Information), adding ENG can initially reduce the levelized cost of storage for LaNi_5_ MH. However, further increases in ENG lead to higher costs due to the resulting decrease in volumetric energy density. Other methods, such as tubes fins and aluminum foam have also been employed.^[^ ^56^ ^]^ The impact of these methods on the thermal properties of MHs and their cost performance requires further investigation. Moreover, we use the energy balance method to evaluate the total amount of heat that must be supplied during the discharging, and we use high‐temperature steam to transfer this heat. However, identifying the most efficient method for large‐scale industrial heat transfer remains a challenge to widespread application of MHs. In addition to the heat supply analyzed in this study, other potential sources for providing heat during discharge include combustion of a portion of the hydrogen released from the storage material. As shown in Note S7 (Supporting Information), this approach would require storing at least 20% more hydrogen to account for combustion needs. Moreover, the cold start challenge must be addressed for this strategy to be effective. Although the overall LCOS is not calculated for this scenario, it is clear that it would be higher than our base case, as a portion of the stored hydrogen would not be available for electricity generation via fuel cells. However, this approach could enable a self‐sustained system without the need for external fuel or electricity. Another theoretical solution stores the heat generated during the charging process (an exothermic reaction) and reuses it during discharge. While promising, the technical feasibility of this approach for large‐scale hydrogen storage, especially for backup power applications with long cycle durations, remains uncertain. The use of phase‐change materials (PCM) has been studied for storing heat,^[^ ^56^, ^57^ ^]^ but the practicality of this approach, considering the massive amount of PCM required and the associated energy costs, requires further investigation.

Additionally, the cyclability and degradation of MHs play vital roles in their competitiveness for hydrogen storage applications. In our base case, we estimate a requirement of 12 cycles per year, totaling ≈360 cycles over a 30‐year lifespan, with a 10% loss of storage capacity. To account for this, we oversize the system by including an additional 10% of MHs in the tank to compensate for the anticipated reduction in hydrogen storage capacity. This degradation rate, however, is likely to vary across different MH materials. To address this variability, we conducted a sensitivity analysis (Figure S9, Supporting Information), evaluating the impact of material degradation on the required MH quantity, volumetric energy density, and LCOS. Despite these modeled projections, accurate experimental testing of MH cyclability and degradation remains essential for reliable assessment of system‐level cost performance.

Kinetics is another critical property that determines the practical application of MHs for hydrogen storage. Intermetallic MHs tends to exhibit favorable thermodynamics and fast kinetics, whereas lightweight hydrides and complex hydrides, which have high storage capacities, often have suboptimal thermodynamics and slow kinetics.^[^ ^58^ ^]^ For long‐duration hydrogen storage, rapid hydrogen absorption is less critical compared to transportation applications, where refueling needs to occur in minutes. The primary goal for stationary hydrogen storage is to safely store hydrogen for extended periods while maximizing volumetric energy density. For MHs with sluggish kinetics, multiple storage tanks can be charged and discharged simultaneously to meet the required hydrogen input rate for fuel cells, allowing sufficient time for each tank to complete its cycle. Thus, a detailed kinetic analysis is not included in this study and but could be considered in future investigations of system‐level storage costs. Extensive research has been conducted to improve absorption and desorption kinetics through alloying, nano‐sizing, and catalysis at the laboratory scale.^[^ ^58^, ^59^, ^60^ ^]^ However, understanding how to scale up these promising MHs and their effects on cost performance is important for future research.

Moreover, for PEM electrolyzers, the H_2_ outlet pressure is ≈30 bar, which is sufficient to avoid the need for a compressor in intermetallic MH‐based hydrogen storage systems. The required inlet pressure for a PEM fuel cell ranges from 2 to 5 bar, an achievable pressure level during desorption from MH storage.^[^ ^30^, ^61^ ^]^ In this study, a desorption pressure limit of 2 bar was employed. However, large‐scale power storage systems may encounter significant pressure drops during hydrogen flow through pipelines, adversely affecting system performance and increasing the LCOS. As detailed in Note S5 (Supporting Information), increasing this limit to 5 bar to account for potential pressure drops results in a rise in LCOS. The exact value of pressure drops and potential hydrogen loss during dispensing depends on the coupling of the MH‐H_2_ storage system with the plant design and specific dispensing distance. These factors were not included in the current model and deserve further investigation to optimize hydrogen storage system design and operation.

Finally, we note that there are numerous emerging hydrogen storage technologies, including include physical adsorption‐based materials such as MOFs and chemical hydrogen carriers like liquid organic hydrogen carriers (LOHCs).^[^ ^1^, ^7^, ^58^, ^62^ ^]^ Storage systems using MOFs for power applications have been studied in detail as MOFs exhibit excellent hydrogen desorption kinetics at moderate temperatures and pressures that make them interesting for small fuel cell applications.^[^ ^63^ ^]^ LOHCs, have been studied extensively for bulk hydrogen storage for transportation, as they are highly compatible with existing liquid fuel infrastructure. However, many LOHC rely on a carbon source or a petroleum based carrier molecule, and require high operating temperatures and involve complex catalytic reactions for hydrogenation and/or dehydrogenation, adding to the complexity of the overall power to power system.^[^ ^64^ ^]^ For example, ammonia, as a hydrogen carrier, offers high volumetric energy density and ease of transportation, though it poses challenges related to toxicity, corrosiveness, and the need for efficient decomposition technologies to release hydrogen effectively.^[^ ^65^ ^]^ Future work is needed to develop a comprehensive comparison of system costs, efficiency, and performance across these diverse hydrogen storage technologies. This includes thoughtful consideration in possible variations in system design and balance of plant, and technical performance requirements at expected operating conditions and cycles.

## 4 Conclusion

In this study, we introduce novel process designs for metal hydride‐based hydrogen storage systems for backup power applications. We calculate their system energy density, footprint and LCOS, benchmarking them against compressed gas hydrogen storage, and set targets for materials development.

We employ a storage tank design with internal cooling tubes, optimized using the “acceptability envelope” method. Coolant or heating steam is circulated through these tubes to facilitate efficient heat transfer during charging and discharging processes. After charging, the storage tank is allowed to cool down to room temperature. Before discharging, the tank is reheated to release the hydrogen at the required operating temperature. We then calculate the capital and operational costs of various equipment using an energy balance method, accounting for the power and energy required to size the equipment across the different operational stages.

For a base case scenario with 96‐h charging, 10 MW discharge for 96 h, and 12 operating cycles per year, intermetallic MHs exhibit higher LCOS compared to physical‐based hydrogen storage but offer significantly higher energy density and a substantial advantage in land footprint, requiring up to 65% less space than 170 bar compressed gas storage. Storing 5% of gas and utilizing waste heat from the fuel cell further reduce the LCOS of intermetallic MHs. However, this approach involves a trade‐off, as the increased storage of gas‐phase hydrogen results in a decrease in system volumetric energy density. Among the intermetallic materials analyzed, TiFe_0.85_Mn_0.05_ at 328 K and 25 bar achieves the lowest LCOS ($0.41/kWh) with a good balance of volumetric energy density (0.65 kWh L^−1^). Complex MH Mg(NH_2_)_2_‐2.1LiH‐0.1KH achieves a lower LCOS than 350 bar compressed gas, primarily due to high hydrogen uptake and reduced capital cost, which offset the increased operational costs.

Extending the charging time reduces the LCOS across all systems by lowering the power requirements for key equipment. Mg(NH_2_)_2_‐2.1LiH‐0.1KH shows the most significant reduction in LCOS due to their higher reliance on heat removal and cooling. Additionally, slower charging further reduces the required footprint for MHs, as fewer cooling tubes are needed in each tank, making MHs more competitive for applications with limited space. Meanwhile, increasing the frequency of annual operating cycles reduces the LCOS for all systems. However, intermetallic MHs experience a more substantial reduction due to their higher capital costs, which benefit from more frequent use. In contrast, complex MH Mg(NH_2_)_2_‐2.1LiH‐0.1KH with lower capital costs are more competitive when the system is used less frequently, making them suitable for long‐duration energy storage.

To further enhance the economic competitiveness of MHs, future research should focus on optimizing thermal management, increasing hydrogen uptake, and reducing MH production costs. These advancements are crucial for realizing the full potential of MH‐based hydrogen storage systems. Our analysis of various power scales and durations indicates that MH‐based hydrogen storage systems offer significant potential for medium power and medium to long‐duration energy storage. Key in enabling this emerging technology is developing scalable manufacturing methods that reduce material waste and generate MH with reliable properties.

## Conflict of Interest

The authors declare no conflict of interest.

## Supporting information



Supporting Information

## Data Availability

The data that support the findings of this study are available from the corresponding author upon reasonable request.
