# Supplementary material for: Technoeconomic Insights into Metal Hydrides for Stationary Hydrogen Storage
Source: Adv Sci (Weinh). 2025 Apr 3;12(21):2415736. doi: 10.1002/advs.202415736 (PMC12140348; doi:10.1002/advs.202415736)
Supplement: Supplementary file 1 — Supporting Information [file ADVS-12-2415736-s001.pdf]

ADVANCED SCIENCE
Open Access

Supporting Information

for *Adv. Sci.*, DOI 10.1002/advs.202415736

Technoeconomic Insights into Metal Hydrides for Stationary Hydrogen Storage

*Xinyi Wang, Peng Peng, Matthew D. Witman, Vitalie Stavila, Mark D. Allendorf and Hanna M. Breunig**

Supplemental information for

Technoeconomic Insights into Metal Hydrides for Stationary Hydrogen Storage

Xinyi Wang[1,2], Peng Peng[1], Matthew D. Witman[3], Vitalie Stavila[3], Mark Allendorf[3], Hanna Breunig[1*]

[1]Lawrence Berkeley National Laboratory, Berkeley, CA 94720, United States

[2]Department of Mechanical Engineering, California State University, Fullerton, Fullerton, CA 92870, United States

[3]Sandia National Laboratories, Livermore, CA 94550, United States

*Corresponding author: hannabreunig@lbl.gov

This PDF file includes:

Figure S1 to S13
Table S1 to S6
Supplementary text

Table S1. Key system-level assumptions and design specifications for the base case stationary hydrogen storage back-up energy system

Key assumptions and system specifications	Values	References
Fuel cell efficiency	50%	Fuel cells fact sheet, 2015[1]; Wei et al.2014[2]
Compressor efficiency	70%	Turton et al., 2018[3]; Sdanghi et al. 2019[4]
Heater efficiency	70%	Aboud et al., 2019[5]
Target discharge duration (h)	96	National Fire Protection Association[6]
Annual discharge duration (h)	1152	This study
Charging time (h)	96	This study
Feed temperature from electrolyser (K)	353	Haug et al., 2017[7]; Brauns et al., 2020[8]
Feed pressure from electrolyser (bar)	2	Haug et al., 2017[7]; Brauns et al., 2020[8]
Fuel cell temperature (K)	353	Wong et al., 2019[9]
Fuel cell pressure (bar)	2	Wong et al., 2019[9]

Table S2. Material properties of metal hydrides

Name	Type	Crystalline density (kg/m^3)	Absorption enthalpy (kJ/mol)	Thermal conductivity (W/m/K)	Heat capacity (kJ/kg/K)	Gravimetric hydrogen capacity (wt%)	Usable hydrogen capacity (wt%)	Reference
TiFe	AB	6500	27.4	1.7	0.45	1.70	1.61	Kempf et al.[10], Mintz et al.[11], Dematteis et al.[12]
$TiFe_{0.85}Mn_{0.05}$	AB	6500	32.5	1. 7	0.45	1.84	1.73	Dematteis et al.[12]
$Ti_{0.95}Zr_{0.05}Mn_{1.55}$-$V_{0.45}Fe_{0.09}$	AB_2	6100	28.4	1	0.50	1.89	1.66	Herbrig et al.[13], Vanhanen et al.[14], Voskuilen et al.[15]
$MnNi_{4.6}Fe_{0.4}$	AB_5	8400	30.5	1.6	0.42	1.48	1.38	Voskuilen et al.[15] Muthukumar et al.[16], Satheesh et al.[17], Satheesh et al.[18]
$Mg(NH_2)_2$-2.1LiH-0.1KH	Complex	1090	40.4	3.8	2.15	4.50	4.07	Allendorf et al.[19,20]

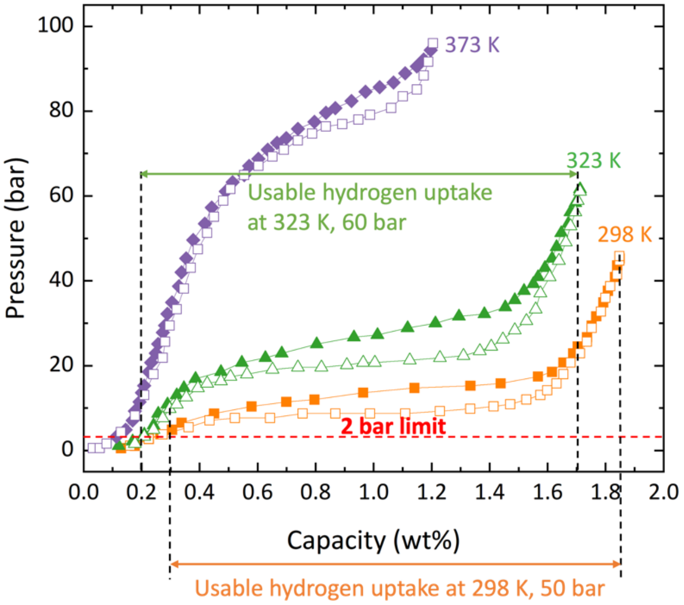

Figure S1. Pressure-composition-temperature curve for an AB_2 metal hydride (MH) ($Ti_{0.95}Zr_{0.05}Mn_{1.55}$-$V_{0.45}Fe_{0.09}$). The usable hydrogen uptake is determined based on temperature and a required output pressure of 2 bar. Data obtained from Purdue Metal Hydride Toolbox Summary[15].

SI Note 1. System-level performance calculations and tank design.

In this study, we calculate the energy and power requirements for each stage based on an energy balance method. The required total hydrogen amount is determined by Equation (1).

$$Capacity = Power(MW) \times 1000 \times \frac{t_{duration}}{LHV} / efficiency \quad (1)$$

In the base case scenario, we assume power is 10 MW, $t_{duration}$ is 96 hours. The lower heating value (LHV) for hydrogen ranges from 33.3 kWh/kg to 33.6 kWh/kg[21]. The efficiency of the system is determined by several factors: fuel cell efficiency (50%) and the release efficiency of various metal hydrides.

For each storage tank, the stored amount of hydrogen is:

$$m_{H2\_tank} = m_{MH_{tank}}/(1 + r_{degrade}) \times m_{H2\_uptake} \tag{2}$$

Where, $m_{MH_{tank}}$ represents the mass of MH per storage tank in kilograms, $r_{degrade}$ is the degradation rate of MH, which we assume to be 10% in our base case. m_{H2_uptake} is the hydrogen uptake per kg of MH.

The number of storage tanks is calculated via Equation (3) and rounded up to an integer:

$$N_{tank} = Capacity/m_{H2\_tank} \tag{3}$$

The total metal hydride mass is given by:

$$m_{MH} = N_{tank} * m_{MH\_tank} \tag{4}$$

To calculate the volume of MHs for each tank, we first determine their bulk density (ρ_{bulk} in kg/m^3) based on the crystalline density ($\rho_{crystal}$) and pellet porosity (ε_p) via Equation (5).

$$\rho_{bulk} = \rho_{crystal} * (1 - \varepsilon_p) \tag{5}$$

Based on the volume of metal hydride per storage tank, we can determine both the volume and mass of the tank. To facilitate the circulation of heating steam and coolant within each storage tank and ensure efficient charging and discharging, we utilize a tank design that incorporates coolant tubes inside each tank. The number and spacing of these cooling tubes within the tank are calculated using the adiabatic form of the metal hydride acceptability envelope in cylindrical coordinates, a model previously developed by Corgnale et al.[22]. Derivation of the acceptability envelope will not be provided here, but the resultant equation, as cited from Corgnale et al. is[22]:

$$\frac{1}{L} \times \left(\frac{k m_{MH\_tank} \Delta T}{-\Delta H_{abs} \rho_{bulk}}\right) = \frac{1}{A \times M_{H2}} \times \left(\frac{m_{H2\_tank}}{\Delta t}\right) \tag{6}$$

Where, L is the characteristic distance between heat transfer surfaces (m), ΔT is the acceptable temperature rise during refueling (K), m_{MH_tank} is the mass of hydride to load (kg), k is thermal conductivity (W/m/K), A is 4 for cylindrical coordinate, $\frac{m_{H2\_tank}}{\Delta t}$ is the required charging rate of hydrogen (kg/s), ΔH_{abs} is heat of reaction (J/mol/H_2), ρ_{bulk} is bulk density (kg/m^3), M_{H2} is the molecular weight of hydrogen (kg/mol). In cylindrical coordinates, the characteristic length L is defined by the inner surface radius and an adiabatic outer surface radius, as described in Equation (7). The value of γ is provided in Equation (8).

$$L = \sqrt{(r_2{}^2 - r_1{}^2)(\frac{\frac{r_2{}^2}{r_1{}^2}}{\gamma} - 1)} \quad (7)$$

$$\gamma = \frac{\frac{r_2{}^2}{r_1{}^2} - 1}{2\ln(\frac{r_2}{r_1})} \quad (8)$$

Utilizing the radius values derived from the aforementioned equations, the volume of a cooling unit cell within the tank can be calculated. The ratio between the volume of the metal hydride and the volume of a cooling unit cell determines the number of cooling tubes required inside a storage tank. Based on the number of tubes, the internal diameter of the tank, and outer dimensions of the storage tank can be estimated using the "Tankinator" model and "Design Tool" developed at the Pacific Northwest National Laboratory (PNNL)[23–25]. The system-level volumetric energy density E_{dens} (kWh/L) can be determined in Equation (9). The detailed results of the tank dimensions, number of tanks and volumetric energy density of selected MHs under optimal operating conditions are presented in Table S3.

To provide a comparison with the base case tank design, we also evaluate an alternative tank configuration as depicted in Figure S2b. In this design, we assume that an external cooling tube surrounds the metal hydride bed. We calculate the system-level levelized cost of storage (LCOS) for each configuration under various power requirements (detailed LCOS calculations are shown in SI Note 2). The alternative configuration demonstrated a lower LCOS due to its simpler tank design as shown in Figure S2c. However, as the power requirement increases, the number of tanks needed also rises linearly (Figure S2d). Utilizing the alternative configuration is impractical for larger-scale applications, as it requires a significantly higher number of tanks compared to the base case. Using a large number of tanks significantly increase maintenance challenges, space requirements, and pose safety and regulatory concerns.

To quantify the effects of tank size on the LCOS and system energy density, we perform a sensitivity analysis using various tank volumes (assuming a constant length of 5 m) for 170 bar compressed gas. The results, depicted in Figure S3, indicate that variations in volume have a minimal impact on the LCOS and system energy density, which range from 0.28 to 0.29 $/kWh, and 0.29 to 0.30 kWh/L respectively.

$$E_{dens} = \frac{m_{H2\_tank} \times LHV}{V_{tank} \times (\frac{10^3 L}{m^3})} \tag{9}$$

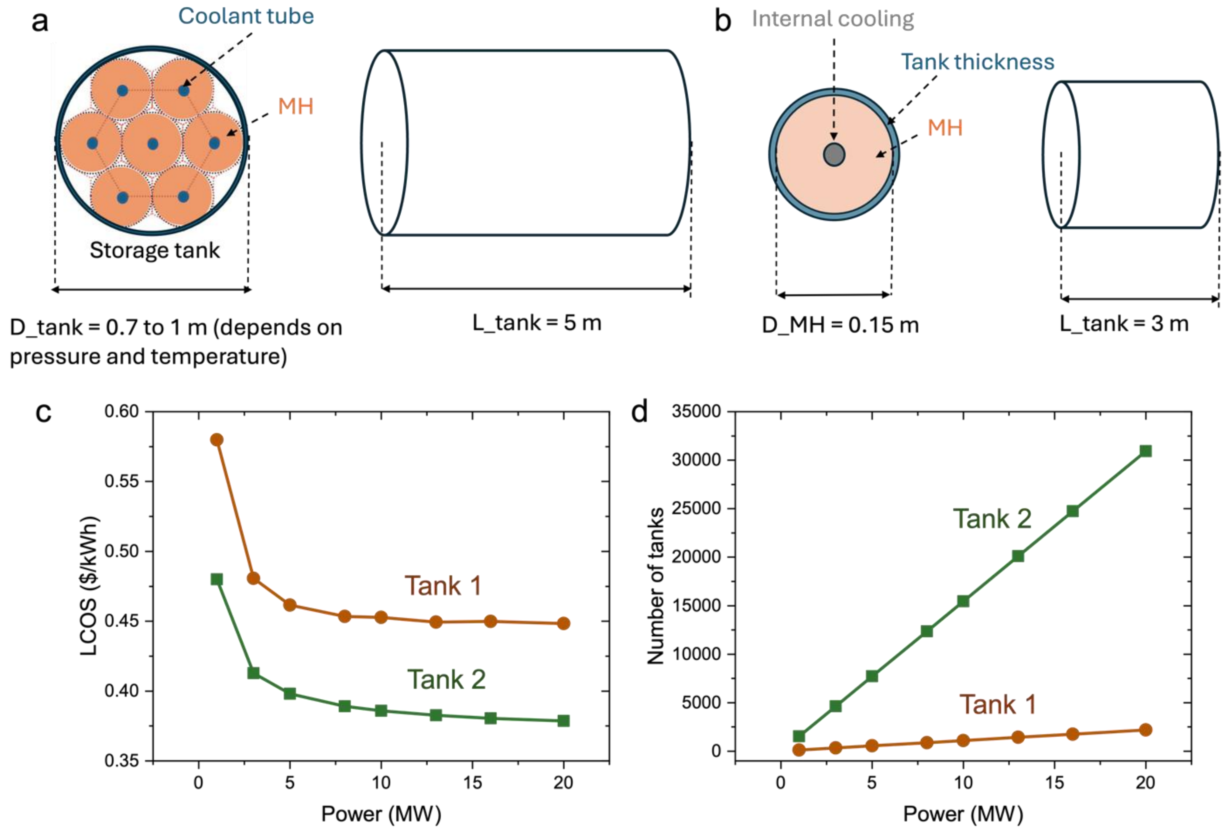

Figure S2. (a) Tank design featuring internal coolant tubes for the base case scenario. (b) Design for a small storage tank with external cooling. The small diameter is chosen to enhance cooling efficiency during the charging process. (c) Levelized cost of storage versus power size for the two tanks designs. (d) The required number of tanks versus power size for the two tank designs.

Table S3. Storage capacity, total MH amounts, storage tank dimensions and system-level volumetric energy density of selected materials.

Conditions	TiFe, 60 bar, 298 K	$TiFe_{0.85}Mn_{0.05}$ 25 bar, 328 K	AB2, 50 bar, 298 K	AB5, 40 bar, 298 K	Complex MH, 80 bar, 511 K	Compressed gas, 350 bar, 298 K	Compressed gas, 170 bar, 298 K
H2 capacity (kg)	60,394	60,902	64,675	61,295	63,315	60,195	60,179
MH mass (kg)	3,917,100	3,633,300	3,788,400	4,547,400	1,547,700	0	0
Number of coolant tubes	68	76	101	68	104	1	1
Number of tanks	1187	1101	1148	1378	469	959	1785
Tank type	Type 1	Type 1	Type 1	Type 1	Type 1	Type 3	Type 1
Tank outer diameter (m)	0.62	0.62	0.68	0.56	1.34	1.00	1.01
Tank length (m)	4.62	4.62	4.68	4.56	5.34	5.10	5.11
Tank volume (m^3)	1.35	1.33	1.62	1.09	6.88	3.72	3.82
Volumetric energy density (kWh/L)	1.27	1.39	1.17	1.37	0.66	0.55	0.30

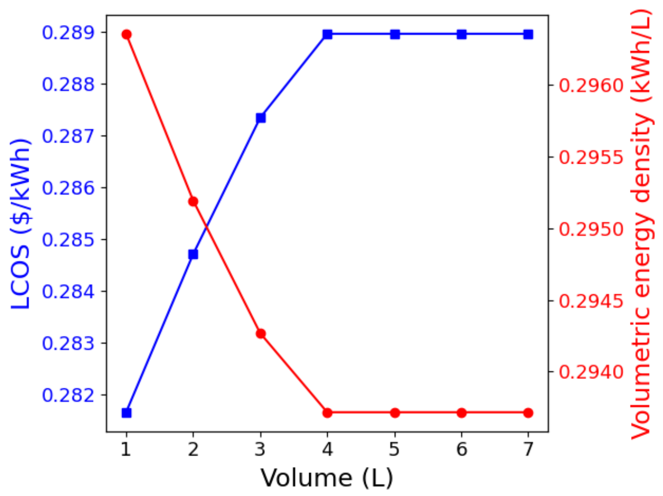

Figure S3. Sensitivity analysis of LCOS versus various storage tank volumes for a 170-bar compressed gas system.

SI Note 2. Techno-economic analysis (TEA)

The total cost of the tank is determined as the product of N_{tank} with the unit cost of each tank determined by the Tankinator model[24]. The cost of metal hydride is given by the product of m_{tank} and the cost of metal hydride. The power and energy required to size and compute the capital and operation costs of various equipment are based on the energy balances between the different operation stages. The enthalpy at each state (j) is calculated via Equation (10)[26].

$$H_j = N_{tank} \times \left(m_{H2-tank} * \left(\Delta H_{abs}\left(T_{j-tank}, P_{j-tank}\right) + \Delta H_{H2}\left(T_{j-tank}, P_{j-tank}\right)\right)\right) + m_{H2-gas} * \Delta H_{H2}(T_{j-gas}, P_{j-gas} + m_{MH-tank} * U_{MH} + m_{wall} * U_{wall} \quad (10)$$

Where ΔH_{abs} is the absorption enthalpy in kJ/kg, U_{MH} and U_{wall} are the specific internal energy of MHs and tank material in kJ/kg respectively. T_{j-tank}, P_{j-tank} are the temperature and pressure in the tank after equilibrium is reached. T_{j-gas}, P_{j-gas} are the temperature and pressure of gas-phase hydrogen outside of the tank, which can be either fed from electrolyzer, or to the fuel cell. The sensible heat for cooling and heating the solids between different states (j and i) is determined using a constant solid heat capacity during the absorption and desorption.

$$U_{j,solid} - U_{i,solid} = \int_{T_i}^{T_j} C_{p,sloid} dt \quad (11)$$

The energy required during the charge and discharge is calculated by the following equation.

$$H_f - H_i = W_s + Q_{\frac{cool}{heat}} + Q_{loss} \quad (12)$$

a) Cost of compressor:

Mechanical Energy of a compressor under isentropic condition (J/kg) is:

$$W_s = N_{tank} * m_{H2-tank} * \frac{k}{k-1} \frac{RT_{in}}{MW_{H2}} ((\frac{P_{out}}{P_{in}})^{\frac{k-1}{k}} - 1) \quad (14)$$

Here, k is the ratio of specific heat, that can be obtained from Turton et al.[3].

Based on the compression power requirements and compressor efficiency, the required mechanical power of compressor pump is (kW):

$$P_{compressor} = \frac{W_s}{1000} * \frac{Total\ amount\ of\ hydrogen\ (kg)}{Charge\ time\ (h) \times 3600} / Efficiency \quad (15)$$

We then calculated the capital cost of compressor:

$$Cap_{compressor} = 10^{K_1 + K_2 \log(P_{compressor}) + K_3 (log P_{compressor})^2} \quad (16)$$

For centrifugal compressor, K_1 is 2.2897, K_2 is 1.3604, K_3 is -0.1027[3].

b) Cost of heater:

The power and energy required for heaters are determined by the following equations. For the heating unit, the cost parameters K_1 is 6.9617, K_2 is -1.48, K_3 is 0.3161[3].

$$Q_{heater} = H_f - H_i - W_s \quad (17)$$

$$P_{heater} = \frac{Q_{heater}}{t_{discharge} * \frac{3600s}{1h} * \eta_{heater}} \quad (18)$$

c) Cost of refrigeration[27]:

We employ a similar method to calculate the required power for the refrigeration unit. The cost is \$800 000 for a refrigeration capacity of 1000 kW, with economy scale factor n of 0.77 across a range from 20 to 5000 kW. For example, for a refrigeration capacity of 3000 kW and 5 °C coolant, the capital cost is:

$$Cap_{ref} = 800000 \times (\frac{3000}{1000})^{0.77} \tag{19}$$

The utility cost of refrigeration coolant is detailed in Table S4[3]. To efficiently remove the heat during the charging process in MHs and enhance the absorption kinetics, we used refrigeration coolant at a low temperature, costing 8.49 \$/GJ.

Table S4. Utility cost of refrigeration coolant at various temperatures

Utility	Cost (\$/GJ)
Moderately low temperature refrigerated water in at T = 5°C and returned at 15°C	4.77
Low temperature available at T = -20°C	8.49
Very low temperature available at T = -50°C	14.12

d) When using historical records for prices information, we adjust the costs to reflect changing economic conditions, such as inflation. This adjustment was made using the following expression[3]:

$$C_2 = C_1 \frac{I_2}{I_1} \tag{20}$$

Where C is the purchased cost, I is the cost index. We use Chemical Engineering Plant Cost Index from 2001 to 2023. Finally, the levelized cost of storage is determined based on the annual storage capacity using Equations (21) and (22). The number of operating years (n), real discount rate (D), tax rate (tax) and the value of depreciation (D_{pv}) are presented in Table S5.

$$CRF = \frac{D(1+D)^n}{(1+D)^n - 1} \tag{21}$$

$$LCOS = (\frac{(\sum Cap) * CRF * (1 - Tax * D_{pv})}{(Annual\ H2\ capacity * (1 - Tax)} + \frac{\sum Annual\ O\&M}{Annual\ H2\ capacity})/LHV_{H2} \tag{22}$$

e) Labor cost

We start with using the following equations from Turton's as the basis for calculation labor costs[3].

$$N_{OL} = \left(6.29 + 0.23N_{np}\right)^{0.5} \tag{23}$$

$$C_{labor} = N_{OL} \times working\ hours\ per\ year\ \times wage \tag{24}$$

Where, N_{OL} represents the number of operators per shift, and N_{np} is the number of nonparticulate processing steps, including compression, heating and cooling. For our hydrogens storage system, each type of unit operation is considered as one piece of equipment. Therefore, N_{np} is 4, accounting for the compressor, storage tank, refrigerator and heater. The system is designed for year-round operation, running 24 hours per day, 365 days per year. The wage rate is set at 36 \$/hour based on data from US Bureau of Labor Statistics[28].

This calculation results in a constant labor cost of \$846,787, which is independent of the power size of the storage system. Consequently, this leads to a higher LCOS for smaller power sizes, as shown in Figure S4a and S4b. However, for a more practical consideration, smaller power size systems store less hydrogen and requires less monitoring. Therefore, we revise our method of calculating labor costs, introducing method 2 as an improvement on method 1. In method 2, the number of equipment considered is based not only on the type of equipment, but also on the actual number of each equipment type and their operating periods. For example, compressors and refrigeration are employed during the charging process, while heaters are used during the discharge process. The labor requirement during charging and discharging is determined by the maximum number of equipment in use, as labor assigned to equipment during charging can also be utilized during discharge. For the storage tank, we assume that every 20,000 kg of stored hydrogen requires one laborer for monitoring. Therefore, during the pure storage period, the number of laborers is based on the total amount of stored hydrogen. Overall, the labor cost is determined by:

$$N_{np\_reaction} = \max[(N_{compressor} + N_{refrigeration} + 2), (N_{heater} + 1)] \tag{25}$$

$$N_{np\_store} = H2\_capacity\ /\ 20000 \tag{26}$$

$$C_{np} = (6.29 + 0.23 \times N_{np\_reaction})^{0.5} \times wage \times n\_cycles \times (t_{ch} + t_{dis}) \tag{27}$$

$$C_{np\_store} = N_{np\_store} \times ((365 \times 24 - n\_cycles \times (t_{ch} + t_{dis})) \times wage \tag{28}$$

From Figure S4b, it is evident that method 2 more effectively accounts for the impact of power size of labor costs.

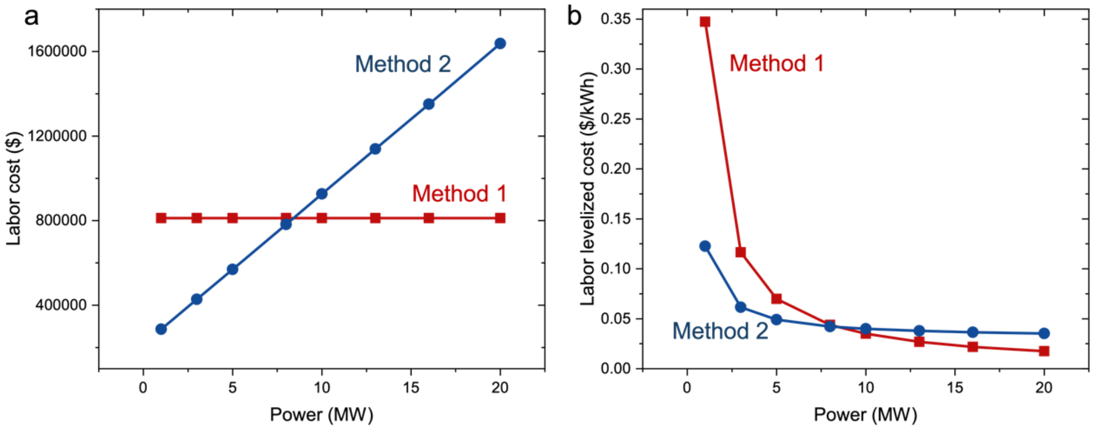

Figure S4. (a) The total labor cost for two different methods versus the power size of the system. (b) Labor levelized cost versus power size of the system for two methods.

f) Insulation cost

The heat transfer between the storage tank and the ambient environment is analyzed. The effects of internal convective heat transfer during charging and discharging are not modelled. The heat loss per unit area from an energy storage tank, specifically through a cylindrical tank wall, can be estimated using the concept of thermal conduction. The basic equation for heat transfer through a cylindrical wall, derived from Fourier's law of heat conduction is given by Equation (29).

$$dQ/_{dt} = 2\pi L_{tank}(T_{storage} - T_{amb})/(\frac{\ln\left(\frac{r_{tank_{out}}}{r_{tank_{in}}}\right)}{k_{tank}} + \frac{\ln\left(\frac{r_{ins_{out}}}{r_{ins_{in}}}\right)}{k_{ins}}) \quad (29)$$

Where k_{tank} and k_{ins} are the thermal conductivities of the storage tank and insulation material. For the storage tank, we use 6061 T6 Al, which has a thermal conductivity of 167 W/m/K. The thermal conductivity of multi-layer vacuum insulations ranges from 0.002 W/m/k to 0.008 W/m/k)[29,30]; for our calculations, we chose 0.004 W/m/k. The cost of insulation is \$30/kg[31]. The total cost of insulation is calculated by:

$$C_{ins} = 30 \times \pi({r_{ins_{out}}}^2 - {r_{ins_{in}}}^2) \times L_{tank} \times \rho_{ins} \times 1.9 \quad (30)$$

Where, the density of vacuum insulation is 210 kg/m^3. 1.9 is the process and installation factor[32]. From Figure S5a, the time required for the system to lose temperature increases with the thickness of insulation layers. Additionally, as indicated by the green line in Figure S5b, the energy cost to

maintain the temperature decreases with increasing insulation thickness. There is a tradeoff between the increased capital cost due to thicker insulation and the decreased energy cost. At a thickness of 0.3 cm, the total capital cost of insulation is minimized. Given the relatively small contribution of insulation to the LCOS, this cost is not included in the LCOS calculations for the main results.

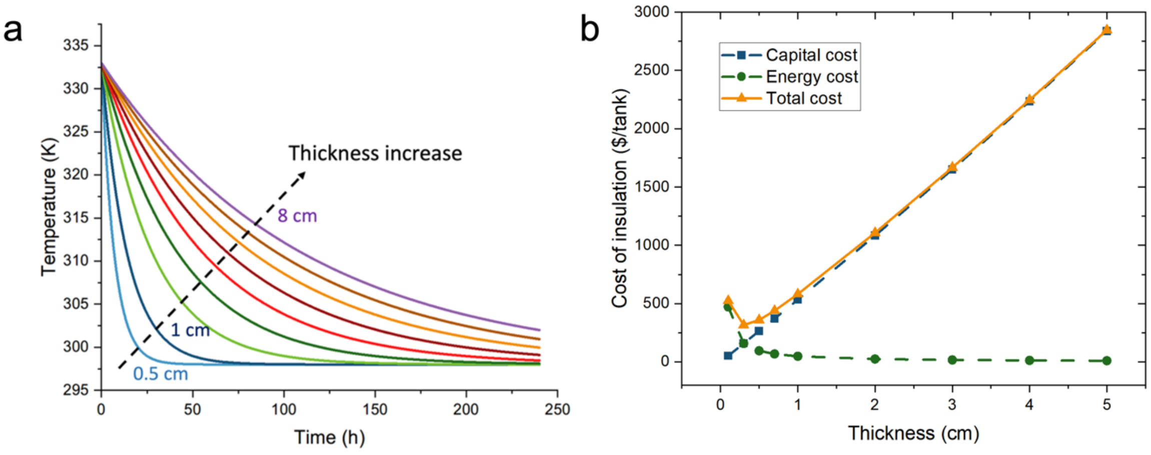

Figure S5. (a) Total time for the storage tank to cool down to room temperature versus insulation thickness. (b) Capital cost of insulation and operational energy cost of maintaining storage temperature with varying insulation thicknesses.

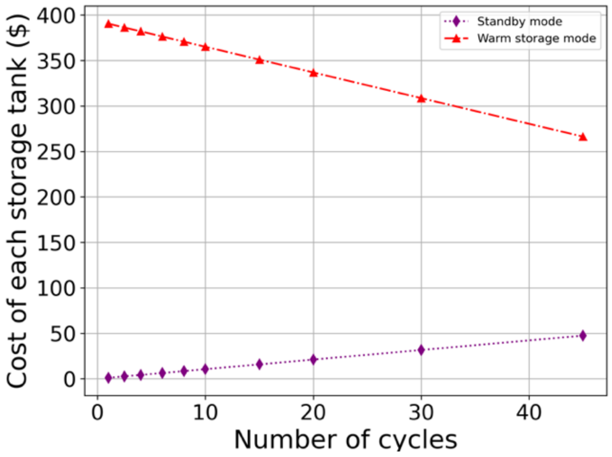

Figure S6. Cost comparison between two process modes: stand-by mode and warm storage mode.

Table S5. Key cost factors for the TEA analysis in this study. Material cost is derived from conversations with alloy manufacturing companies.

Cost factor	Value	Reference
Storage tank lifetime (years)	30	This study
Equipment lifetime (years)	15	This study
Real discount rate	0.07	PNNL[23]
Present value of depreciation	0.83	PNNL[23]
MH cost ($/kg)	20	This study
6061 Al tank cost ($/kg)	4.45	Tankinator[24]
Land cost ($/acre)	70,000	Industrial land values[33]
Electricity cost ($/kWh)	0.067	PNNL[23]
Labor wage ($/h)	36	U.S. Bureau of Labor Statistics[28]
Maintenance (%)	3%	PNNL[23]
CEPCI 2001	397	CEPCI[34]
CEPCI 2007	525.4	CEPCI[34]
CEPCI 2023	798.7	CEPCI[34]
Installation (%)	12%	NREL[35–37]
Site preparation (%)	2%	NREL[35,36]
Engineering and Design (%)	8%	NREL[35,36]
Project contingency (%)	15%	NREL[35,36]
Up-front permitting costs (%)	15%	NREL[35,36]

g) Land footprint

The total land footprint depends on the size of storage tank, the number of storage tanks, and the safety distance based on the operation pressure. The safety distance in this study is derived from the data provided by Jeffrey LaChance et al[38]. and can be calculated using Equation (31). The number of storage tanks installed along the y and z directions are 4 and 9, respectively. For each tank along y and z directions, we assumed a gap distance of 1 meter. The number of tanks along x direction is calculated based on the total number of tanks for the storage system. For every four tanks along the x direction, we assume 1-meter gap distance for regular checking and maintenance. The detailed footprint of the storage system is shown in Figure S7.

$$SD = 2.850897 + 0.04141 \times P - 0.000033 \times P^2 \quad (31)$$

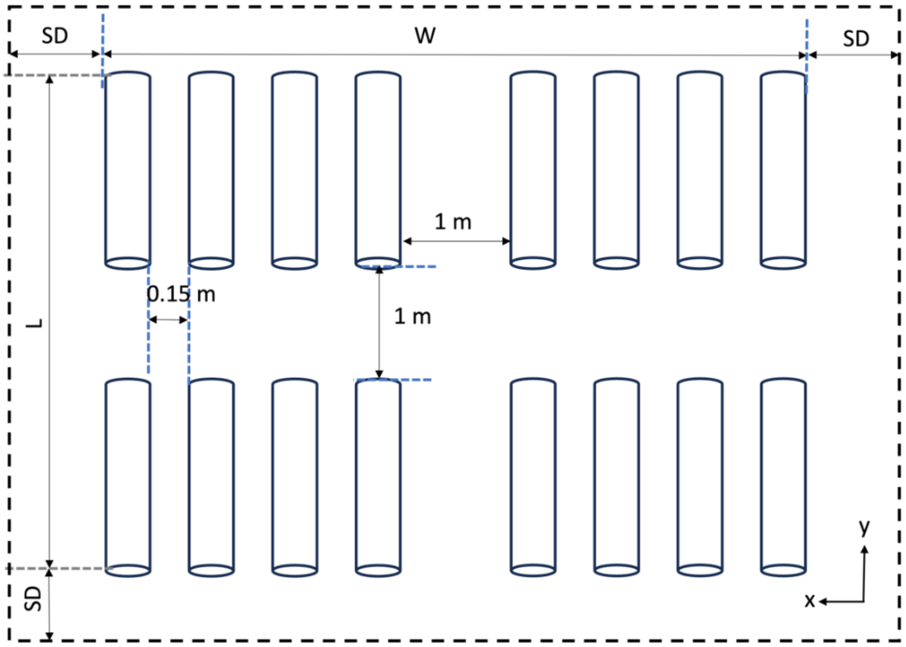

Figure S7. Top view of the footprint of the storage system

h) Compressed H2 storage

In this study, both the system-level performance and TEA for the compressed hydrogen storage are modeled using the same methodology as previously described, excluding the MH contents. Type I tanks are used for compressed hydrogen gas storage under 170 bar conditions. For

compressed hydrogen gas storage under 350 bar, the type of storage tank is changed from Type I to Type III, which is suitable for the high-pressure conditions.

i) Installed capital cost

The levelized cost of storage presented in the manuscript assesses the cost effectiveness of hydrogen storage system over their entire lifecycle. It considers not only capital costs but also operating expenses, as well as economic factors such as taxes, discount rates, and depreciation. In addition to levelized cost of storage, we also calculate the total installed capital cost, as shown in Figure S8, excluding the economic effects of depreciation in the annualized values. Similar to our findings for LCOS, the cost of MHs is a significant component of the capital cost. Complex MH achieves the lowest capital cost due to its high hydrogen capacity, which reduces the amount of MH material required. Interestingly, while the capital cost difference between MHs and compressed gas is substantial – using $TiFe_{0.85}Mn_{0.05}$ as an example, its capital cost in the base case scenario is $550/kg higher than that of 350 bar compressed gas – their LCOS values are much closer, with only a $0.009/kWh difference. This is primarily because MH systems require lower operational costs for compression. Additionally, MH system have lower direct and indirect costs associated with installation and safety. The large capital cost difference can be mitigated by increasing the number of cycles, as spreading the initial capital investment over more cycles effectively lowers the per-cycle capital cost. However, increasing the usage frequency will not affect operational costs, as those are recurring expenses linked to the energy needed for each individual storage and retrieval process, regardless of how often the system is used.

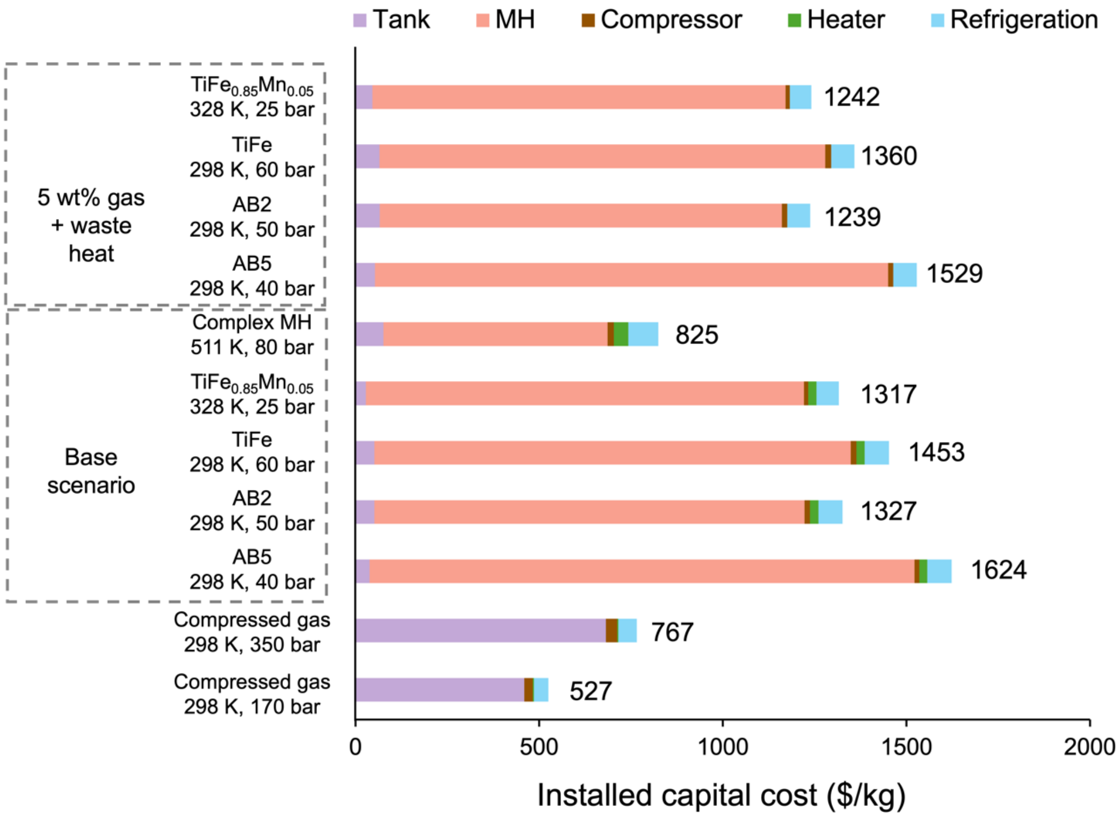

Figure S8. Installed capital cost breakdown for selected MHs hydrogen storage in two scenarios compared with compressed gas hydrogen storage.

SI Note 3. Material cyclability

In this study, we assume the system operates 12 cycles per year over a project duration of 30 years, resulting in a total of 360 cycles. For each metal hydride, we account for a 10% hydrogen capacity degradation by adding an additional 10% of the required metal hydride to the system. This assumption is valid for 360 cycles based on the cyclability data we obtained from the literature. The following summary synthesizes the findings from multiple studies on the cyclic stability and hydrogen storage capacity of various metal hydrides subjected to cycling processes.

A. TiFe: TiFe was cycled under pressures of 32-34 bars for up to 13000 times at the temperature ranging from -7 °C to 110 °C. However, cycling at elevated pressures beyond the second plateau led to a decrease in storage capacity[39]. The dynamic absorption -desorption isotherms show an inherent cyclic instability in the dihydride phase of TiFe. This instability is

characterized by a gradual increase in the plateau pressures over subsequent cycles, which reduces the effective usable hydrogen capacity[40]. After few hundreds cycling, the desorption isotherm becomes increasingly distorted, shifting toward higher pressures and lower hydrogen content in the γ-β region. This reduces the effective hydrogen storage capacity and stabilizes only after hundreds of cycles, whereupon it becomes relatively insensitive to further cycling. The phenomenon is attributed to lattice strain and defects introduced by significant volume changes during the hydriding-dehydriding processes, which promote the formation of intermediate hydrogen phases that are not fully reversible[41]. Additionally, the shapes of the PCT curves for TiFe change with the number of thermal cycles, and a gradual decrease in hydrogen storage capacity is observed after approximately 1,000 cycles due to the suppression of the γ-phase formation in the hydride[42].

B. TiFe variants: The $TiFe_{0.8}Mn_{0.15}$ alloy exhibited no loss in capacity after 20000-30000 cycling through a lower pressure plateau[43]. And the $TiFe_{0.8}Ni_{0.2}$ alloy was reported having15% degradation in hydrogen capacity after 45,000 cycles in the temperature and the pressure ranges of 20 °C–185 °C and 0.5–9 bar, respectively.

C. Mg based MH: The cyclic stability of MgH_2-5wt%V remains up to 2000 cycles, with no obvious changes in isotherms and or material disintegration even at a hydrogen content of 5wt%[44]. Ni-doped Mg and Mg_2CoH_5 show almost stable performance after 800 cycles with minimal fluctuations in the hydrogen capacity[45]. However, Dehouche et al. reported a 15% decrease in hydrogen capacity after 2100 cycles for nanocrystalline Mg_2Ni, attributed to the formation of the non-hydride forming $MgNi_2$ phase during cycling[46].

D. AB5 MHs: All four AB5 materials degraded during extended thermal cycling. For example, $LaNi_{4.7}Al_{0.3}$ shows an initial degradation rates of approximately $1.2*10^{-4}$ H/M per cycle when cycled between 100 °C and 205 °C at pressures of 1.4 to 2.1 MPa. The degradation rate increases at higher temperatures and pressures, reducing plateau length and increasing its slope and pressure. The degradation mechanism of the AB_5 alloys is related to the disproportionation of the reversible hydrides into more stable hydrides and metallic Ni. It was observed a decrease in the kinetics of hydrogen absorption after 4300 cycles, but no loss in the hydrogen capacity of Mg – 2 at%Ni. The reversible system capacity of that alloy decreased by 36% after 4300 cycles due to the slower kinetics. In addition, the alloy $Ti_{0.98}Zr_{0.02}V_{0.43}Fe_{0.09}Cr_{0.05}Mn_{1.5}$ (HWT 5800) has undergone 42,400 cycles without degradation[47]. The $LaNi_{4.25}Al_{0.75}$ alloy, after 1000

absorption/desorption cycles, showed no significant changes in hydrogen storage capacity, though an increase in hysteresis loss and degradation in hydrogen absorption kinetics was observed[48]. Further studies revealed that alloys like $LaNi_5$, $LaNi_{4.7}Al_{0.3}$, and $MmNi_{4.5}Al_{0.5}$ alloys recover hydrogen absorption content after drastic reductions in the first cycle but experience gradual capacity decreases with extended cycling. The substitution of Al atoms in these alloys seems to reduce oxygen absorption, potentially improving stability by limiting atomic exchange. The $MmNi_{4.2}Al_{0.8}$ alloy showed almost unchanged storage capacity, plateau pressures, and desorption kinetics after 1,000 cycles, with only a minor increase in hysteresis loss at elevated temperatures.

The degradation rate of MHs can vary significantly across different materials. To quantify its impact on system efficiency and cost, we varied the degradation rate for each MH evaluated in this study from 0% to 70%, with 10% degradation representing the base case scenario in the main manuscript. For each case, we calculated the required MH quantities (Table S6 shows the upfront mineral demand for ten 10 MW backup power facilities as an example), global warming potentials, volumetric energy efficiency, and levelized cost of storage (LCOS) as shown in Figure S9. As presented in the figure, the required amounts of MHs increase with higher material degradation rates. The global warming potential (GWP), described in Note 8, follows a similar trend, as increased raw material production leads to higher CO_2 emissions. Among all the MHs evaluated, TiFe-based MHs exhibit the highest overall GWP, primarily due to the large quantities of titanium used (see SI figure S13). Rising degradation rates also results in a continuous decline in volumetric energy efficiency and an increase in LCOS. This trend is due to the need for larger amounts of MHs to compensate for reduced hydrogen absorption capacity, ensuring consistent hydrogen storage and energy output. For all interstitial MHs, the volumetric energy density remains higher than that of compressed gas at 350 bar, even at degradation rates up to 70%. However, higher degradation rates reduce their cost-effectiveness compared to 350 bar compressed gas. In the case of the complex MH $Mg(NH_2)_2$–2.1LiH–0.1KH, its volumetric energy density remains lower than that of 350 bar compressed gas when degradation exceeds 30%, and a lower LCOS is only achieved when degradation is below 15%. These findings highlight the need for material innovations that improve cyclability and reduce degradation.

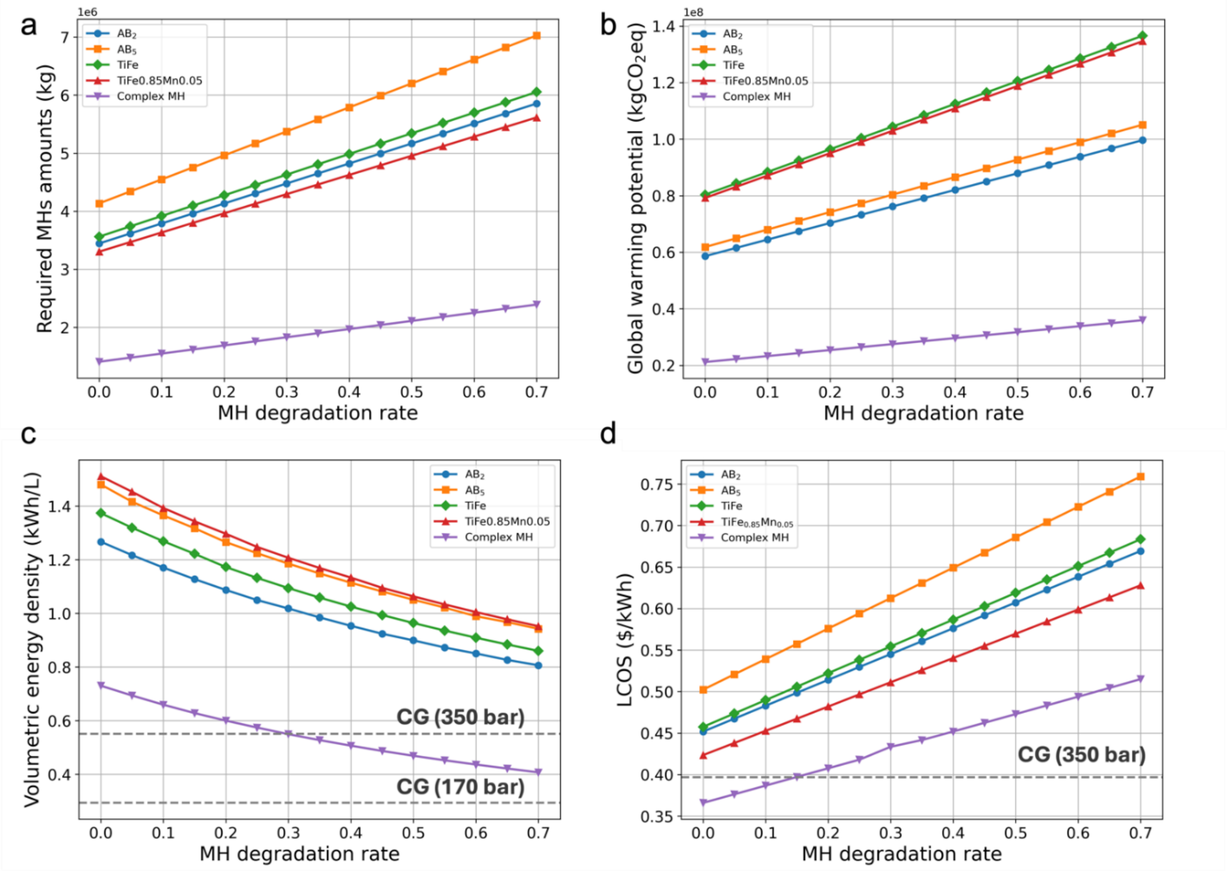

Figure S9. (a-d). Required amounts of MHs, global warming potential, volumetric energy density and LCOS of various MHs as a function of degradation rate, respectively. A degradation rate of 0.1 indicates that 10% additional MHs is required in the storage system to compensate the loss in absorption capacity.

Table S6: Mineral requirement in year 1 for ten 10 MW power facilities, shown as a percentage of individual mineral global production in the year 2023.

Composition	Degradation	Percent of global production for: 10 facilities in 1 year								
		Ti	Fe	Mn	V	Zr	Ni	Mg	Li	K
TiFe0.85Mn0.05	0	0.18%	0.00%	0.00%	0.00%	0.00%	0.00%	0.00%	0.00%	0.00%
TiFe0.85Mn0.05	0.05	0.18%	0.00%	0.00%	0.00%	0.00%	0.00%	0.00%	0.00%	0.00%
TiFe0.85Mn0.05	0.1	0.19%	0.00%	0.01%	0.00%	0.00%	0.00%	0.00%	0.00%	0.00%
TiFe0.85Mn0.05	0.15	0.20%	0.00%	0.01%	0.00%	0.00%	0.00%	0.00%	0.00%	0.00%
TiFe0.85Mn0.05	0.2	0.21%	0.00%	0.01%	0.00%	0.00%	0.00%	0.00%	0.00%	0.00%
TiFe0.85Mn0.05	0.25	0.22%	0.00%	0.01%	0.00%	0.00%	0.00%	0.00%	0.00%	0.00%
TiFe0.85Mn0.05	0.3	0.23%	0.00%	0.01%	0.00%	0.00%	0.00%	0.00%	0.00%	0.00%
TiFe0.85Mn0.05	0.35	0.24%	0.00%	0.01%	0.00%	0.00%	0.00%	0.00%	0.00%	0.00%
TiFe0.85Mn0.05	0.4	0.25%	0.00%	0.01%	0.00%	0.00%	0.00%	0.00%	0.00%	0.00%
TiFe	0	0.18%	0.00%	0.00%	0.00%	0.00%	0.00%	0.00%	0.00%	0.00%
TiFe	0.05	0.19%	0.00%	0.00%	0.00%	0.00%	0.00%	0.00%	0.00%	0.00%
TiFe	0.1	0.20%	0.00%	0.00%	0.00%	0.00%	0.00%	0.00%	0.00%	0.00%
TiFe	0.15	0.21%	0.00%	0.00%	0.00%	0.00%	0.00%	0.00%	0.00%	0.00%
TiFe	0.2	0.22%	0.00%	0.00%	0.00%	0.00%	0.00%	0.00%	0.00%	0.00%
TiFe	0.25	0.22%	0.00%	0.00%	0.00%	0.00%	0.00%	0.00%	0.00%	0.00%
TiFe	0.3	0.23%	0.00%	0.00%	0.00%	0.00%	0.00%	0.00%	0.00%	0.00%
TiFe	0.35	0.24%	0.00%	0.00%	0.00%	0.00%	0.00%	0.00%	0.00%	0.00%
TiFe	0.4	0.25%	0.00%	0.00%	0.00%	0.00%	0.00%	0.00%	0.00%	0.00%
Ti0.95Zr0.05Mn1.55V0.45Fe0.09	0	0.10%	0.00%	0.09%	4.84%	0.06%	0.00%	0.00%	0.00%	0.00%
Ti0.95Zr0.05Mn1.55V0.45Fe0.09	0.05	0.11%	0.00%	0.09%	5.08%	0.06%	0.00%	0.00%	0.00%	0.00%
Ti0.95Zr0.05Mn1.55V0.45Fe0.09	0.1	0.12%	0.00%	0.10%	5.32%	0.07%	0.00%	0.00%	0.00%	0.00%
Ti0.95Zr0.05Mn1.55V0.45Fe0.09	0.15	0.12%	0.00%	0.10%	5.56%	0.07%	0.00%	0.00%	0.00%	0.00%
Ti0.95Zr0.05Mn1.55V0.45Fe0.09	0.2	0.13%	0.00%	0.11%	5.81%	0.07%	0.00%	0.00%	0.00%	0.00%
Ti0.95Zr0.05Mn1.55V0.45Fe0.09	0.25	0.13%	0.00%	0.11%	6.05%	0.08%	0.00%	0.00%	0.00%	0.00%
Ti0.95Zr0.05Mn1.55V0.45Fe0.09	0.3	0.14%	0.00%	0.12%	6.29%	0.08%	0.00%	0.00%	0.00%	0.00%
Ti0.95Zr0.05Mn1.55V0.45Fe0.09	0.35	0.14%	0.00%	0.12%	6.53%	0.08%	0.00%	0.00%	0.00%	0.00%
Ti0.95Zr0.05Mn1.55V0.45Fe0.09	0.4	0.15%	0.00%	0.13%	6.77%	0.08%	0.00%	0.00%	0.00%	0.00%
MnNi4.6Fe0.4	0	0.00%	0.00%	0.03%	0.00%	0.00%	0.89%	0.00%	0.00%	0.00%
MnNi4.6Fe0.4	0.05	0.00%	0.00%	0.03%	0.00%	0.00%	0.94%	0.00%	0.00%	0.00%
MnNi4.6Fe0.4	0.1	0.00%	0.00%	0.04%	0.00%	0.00%	0.98%	0.00%	0.00%	0.00%
MnNi4.6Fe0.4	0.15	0.00%	0.00%	0.04%	0.00%	0.00%	1.03%	0.00%	0.00%	0.00%
MnNi4.6Fe0.4	0.2	0.00%	0.00%	0.04%	0.00%	0.00%	1.07%	0.00%	0.00%	0.00%
MnNi4.6Fe0.4	0.25	0.00%	0.00%	0.04%	0.00%	0.00%	1.12%	0.00%	0.00%	0.00%
MnNi4.6Fe0.4	0.3	0.00%	0.00%	0.04%	0.00%	0.00%	1.16%	0.00%	0.00%	0.00%
MnNi4.6Fe0.4	0.35	0.00%	0.00%	0.04%	0.00%	0.00%	1.21%	0.00%	0.00%	0.00%
MnNi4.6Fe0.4	0.4	0.00%	0.00%	0.05%	0.00%	0.00%	1.25%	0.00%	0.00%	0.00%
Mg(NH2)2-2.1LiH-0.1KH	0	0.00%	0.00%	0.00%	0.00%	0.00%	0.00%	0.47%	1.48%	0.00%
Mg(NH2)2-2.1LiH-0.1KH	0.05	0.00%	0.00%	0.00%	0.00%	0.00%	0.00%	0.50%	1.55%	0.00%
Mg(NH2)2-2.1LiH-0.1KH	0.1	0.00%	0.00%	0.00%	0.00%	0.00%	0.00%	0.52%	1.63%	0.00%
Mg(NH2)2-2.1LiH-0.1KH	0.15	0.00%	0.00%	0.00%	0.00%	0.00%	0.00%	0.54%	1.70%	0.00%
Mg(NH2)2-2.1LiH-0.1KH	0.2	0.00%	0.00%	0.00%	0.00%	0.00%	0.00%	0.57%	1.77%	0.00%
Mg(NH2)2-2.1LiH-0.1KH	0.25	0.00%	0.00%	0.00%	0.00%	0.00%	0.00%	0.59%	1.85%	0.00%
Mg(NH2)2-2.1LiH-0.1KH	0.3	0.00%	0.00%	0.00%	0.00%	0.00%	0.00%	0.61%	1.92%	0.00%
Mg(NH2)2-2.1LiH-0.1KH	0.35	0.00%	0.00%	0.00%	0.00%	0.00%	0.00%	0.64%	2.00%	0.00%
Mg(NH2)2-2.1LiH-0.1KH	0.4	0.00%	0.00%	0.00%	0.00%	0.00%	0.00%	0.66%	2.07%	0.00%

SI Note 4. Effects of adding ENG

Adding expanded natural graphite (ENG) to MHs is a common method to enhance their thermal properties. We want to understand its effect on energy density and levelized cost of storage (LCOS). $LaNi_5$ is selected as an example MH due to its low thermal conductivity. The relationship between thermal conductivity and the mass fraction of graphite is provided by Kim et al.[49] And we assume a linear combination of the heat capacity between graphite and MHs.

$$Cp_{efficient} = \epsilon Cp_{graphite} + (1 - \epsilon)Cp_{MH} \tag{32}$$

Where ϵ is the mass fraction of graphite.

The added ENG will occupy space within the storage tank. If we aim to store the same amount of MHs (3000 kg) in each tank, adding ENG will initially lead to a larger volume due to its space requirement. However, as more graphite is added, the thermal conductivity improves, reducing number of cooling tubes in each tank, which, in turn, decreases the overall volume. This tradeoff in storage tank volume is illustrated in Figure S10a. There is a significant reduction in volume when ENG is added up to 0.05%; beyond this point, further additions have limited impact on reducing thermal conductivity. Instead, the increase in volume due to additional space required by ENG outweighs the reduction in volume from fewer cooling tubes. A similar trend is observed for LCOS, as shown in Figure S10b. While the incorporation of ENG significantly decreases LCOS initially, further increases in ENG result in higher LCOS due to the additional costs associated with the larger storage tanks. It is important to acknowledge that increasing thermal conductivity by adding ENG will enhance the absorption reaction kinetics, which could impact the overall system cost. However, this effect is not accounted for in the current analysis.

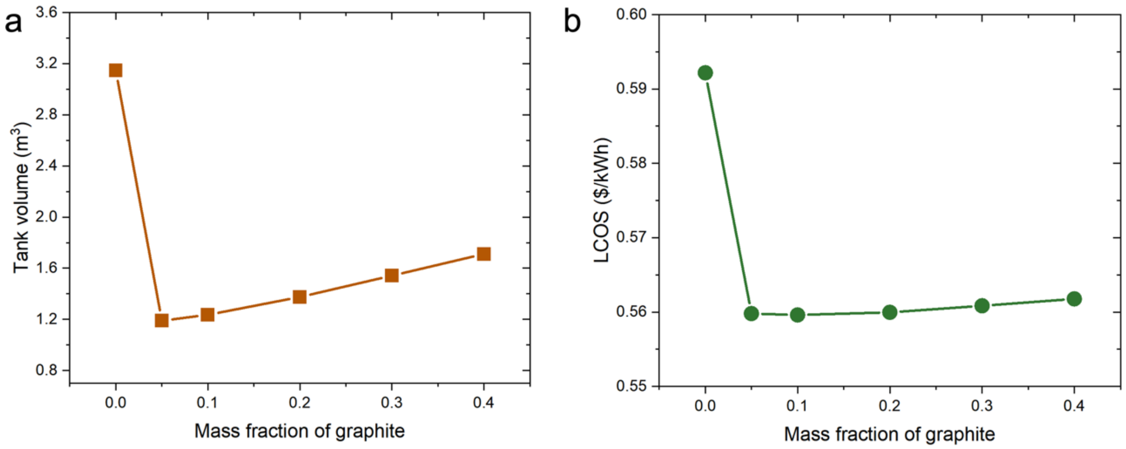

Figure S10. (a) (b) Tank volume and LCOS as a function of mass fraction of graphite in LaNi5, respectively.

SI Note 5. Pressure drop effect

Typically, the hydrogen desorption back pressure ranges from 2 to 5 bar, which is sufficient for transporting hydrogen through pipelines to end-users, such as those utilizing fuel cells. In our base case scenario, we use 2 bar as the desorption pressure limit. However, large-scale power storage systems may experience pressure drops during hydrogen flow through pipelines, which can affect the cost performance. To maintain a 2 bar input pressure for fuel cells, the hydrogen pressure existing the storage system must be higher to account for the pressure drop that occurs during transportation between storage tanks and equipment. While a detailed analysis for pressure drop depends on the specific layout design of the storage system and is beyond the scope of this study, we assume that to maintain a 2 bar input pressure, the output pressure of hydrogen needs to be 5 bar. Figure S11 presents the LCOS and volumetric energy density of various MHs under this scenario. At a same desorption temperature, an increase in back pressure results in a decrease in usable hydrogen content[50]. The reduction in usable hydrogen content increases the LCOS and lowers the energy density. For some MHs with inherently low-pressure levels, raising the output pressure to 5 bar significantly reduces the amount of hydrogen that can be desorbed, thereby increasing the overall LCOS. For certain industrial applications that require higher desorption pressures, increasing the desorption temperature may help maintain a high level of deliverable hydrogen.

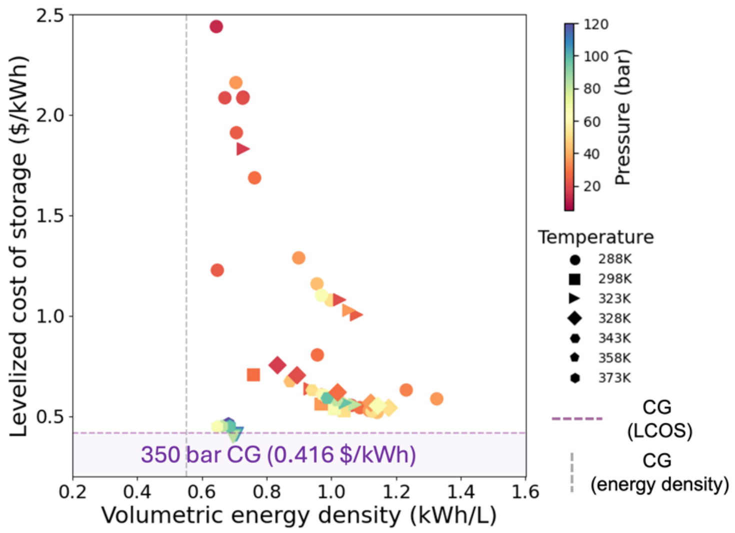

Figure S11. The LCOS and volumetric energy density of materials with a 5-bar back pressure.

SI Note 6. Cost estimates for electrolyzers and fuel cells

We further integrate the storage system costs with the costs of electrolysis and fuel cells for hydrogen generation and utilization to estimate the total system cost. For this analysis, we calculate the capital cost normalized to the delivered power in $/kW, following the method provided in our previous paper[26]. The efficiency for the commercialized alkaline water electrolyzer ranges from 59% to 70%, while the efficiency of the near-commercialized and commercialized proton exchange membrane (PEM) electrolyzers is between 60%–80%[51–53]. To ensure a conservative estimate, we use an efficiency value of 60% for the calculation of upstream electrolyzer costs. The capital costs for the required electrolyzers are obtained from Yates et al.[54] For the fuel cell, we assume a direct manufacturing cost of $200/kWe output[2].

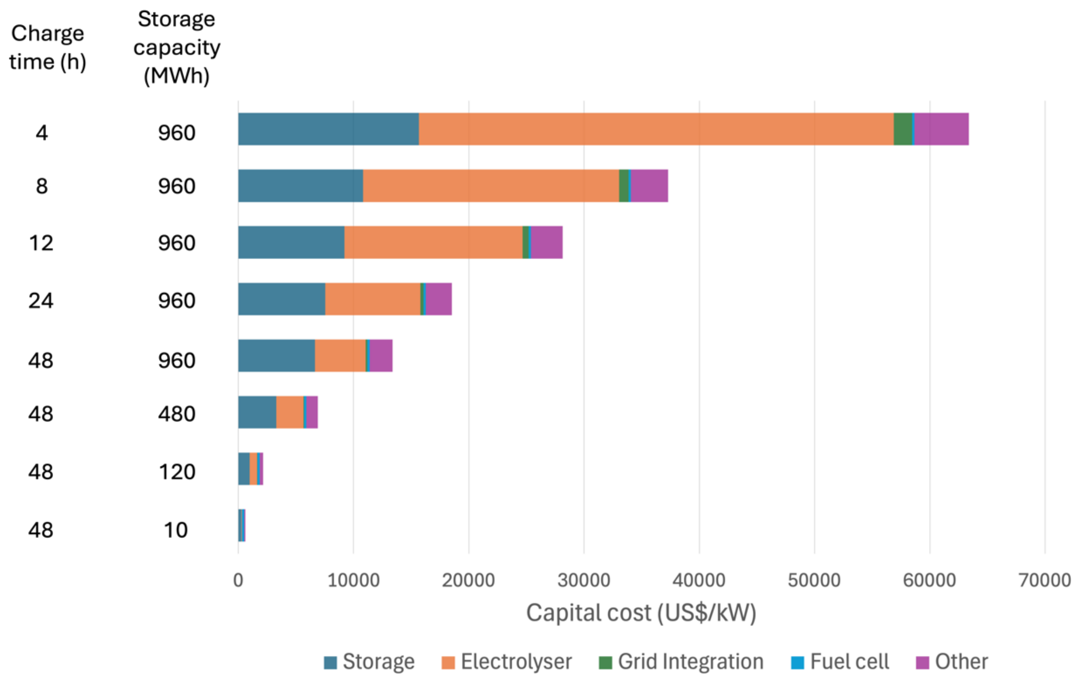

Figure S12. Breakdown of the capital system costs under different storage capacities.

SI Note 7. Hydrogen combustion

Combusting hydrogen to provide heat for hydrogen desorption in MHs is an efficient and sustainable approach to managing the thermal requirements of the system. Hydrogen combustion

produces clean energy with water as the only byproduct, aligning with the overall goal of maintaining a low-carbon footprint. Additionally, using hydrogen for heating ensures that the energy source is consistent and readily available within the system, eliminating the need for external energy inputs. The amount of hydrogen required for combustion to achieve sufficient heat for desorption is dependent on the enthalpy of the MHs and efficiency of equipment, as given in Equation 33. Assuming an enthalpy of 30 kJ/mol and considering both combustion and heat exchange efficiencies at 80%, approximately 20% of the hydrogen must be combusted to meet the thermal demands. While this approach enhances the system's sustainability by reducing reliance on external energy sources, it also increases the overall cost. This cost arises from the need to store 20% more hydrogen, which is not utilized for power generation but instead for heating purposes. Although hydrogen combustion is generally clean, initiating combustion in a cold start scenario may pose challenges. To address this, a small reserve of gaseous hydrogen or an alternative fuel could be used to achieve the necessary reaction temperature. Once the system reaches the appropriate temperature, hydrogen desorption commences, allowing a portion of the released hydrogen to be redirected to a burner[55].

$$x\% = \frac{\Delta H\left(\frac{kJ}{mol}\right)}{241.9/(\eta_{burner} \times \eta_{heat\_exchange})} \tag{33}$$

Where 241.9 kJ/mol is the combustion value of hydrogen, $\eta_{burner}, \eta_{heat\_exchange}$ are the efficiency of burner and heat exchangers, respectively.

SI Note 8. Material life cycle considerations

Figure S13 illustrates two important life cycle impact factors for metal hydride production: the fossil resource factor and 100-year global warming potential (GWP). Like mineral resource factor, fossil resource factor gives an indication of potential damage to resource availability for coal, natural gas, and oil. GWP is also a mid-point factor that gives an indication of radiative forcing from greenhouse gas emissions over a period of time. Impact factors are derived in OpenLCA software using the ecoinvent3.5 database and by averaging GWP results from three reputable impact assessment methods: TRACI 2.1; ReCiPe midpoint (E); IPCC2021. Values are generated using the weight percentage of raw mineral in each MH, and a representative value for ferronickel

chromium alloy synthesis (0.55 kg-CO_2eq/kg alloy). Values are presented for relevant individual elements, as well as the corresponding MHs evaluated in this study. Lithium (Li) from lithium-chloride electrolysis, and titanium (Ti) exhibit notably high GWP and fossil resource factors, indicating the substantial environmental burden associated with their extraction and production processes. This is primarily due to the energy-intensive methods required for their refinement and the scarcity of high-grade ores. In contrast, elements such as vanadium (V), approximated from leaching processes in steel making, and iron (Fe) from sorted and pressed iron scrap, show minimal impacts in both categories, reflecting their lower environmental footprint. In the case of V, it is a byproduct of existing industries and has relatively low competing uses. For the MHs, TiFe-based hydrides show higher GWP and fossil resources factors compared to other MHs, mainly due to the presence of titanium. Conversely, AB_2, AB_5 and complex MHs show moderate environmental impacts in terms of both GWP and fossil resource. This intermediate performance can be attributed to a balanced composition of elements with varying environmental burdens, leading to a more favorable sustainability profile compared to TiFe-based hydrides.

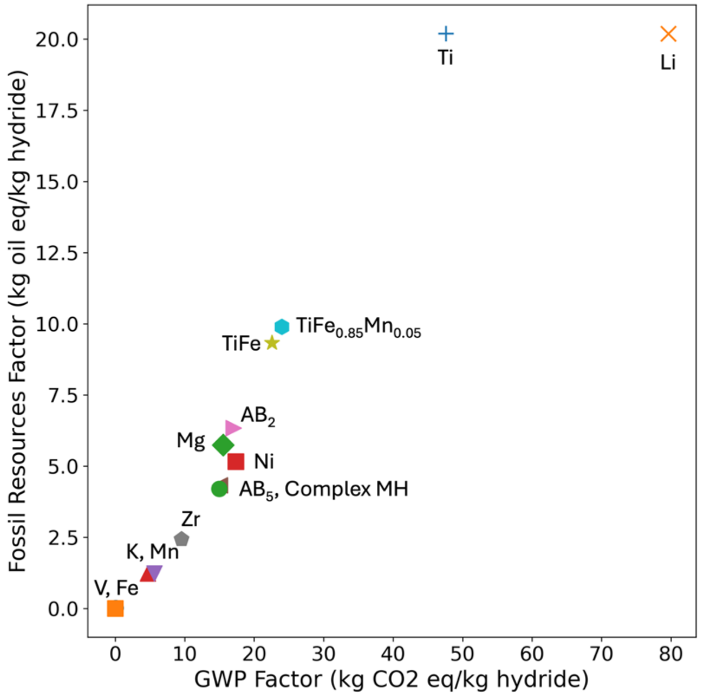

Figure S13. The average global warming potential factor and fossil resources factor for individual elements and MHs.

Reference

1. U.S Department of Energy. FUEL CELLs Fact Sheet. (2015).

2. Max Wei, Timothy Lipman, Ahmad Mayyas, Joshua Chien, Shuk Han Chan, David Gosselin, Hanna Breunig, Michael Stadler, Thomas McKone, Paul Beattie, Patricia Chong, Whitney G. Colella, B. D. J. A Total Cost of Ownership Model for Low Temperature PEM Fuel Cells in Combined Heat and Power and Backup Power Applications. *Lawrence Berkeley Natl. Lab.* (2014).

3. Richard Turton, J. A. S. *Analysis, Synthesis and design of chemical processes*. (2018).

4. Sdanghi, G., Maranzana, G., Celzard, A. & Fierro, V. Review of the current technologies and performances of hydrogen compression for stationary and automotive applications. *Renew. Sustain. Energy Rev.* **102**, 150–170 (2019).

5. Aboud, S. A., Altemimi, A. B., Al-hiiphy, A. R. S., Yi-chen, L. & Cacciola, F. A Comprehensive Review on Infrared Heating. *Molecules* **2**, 1–20 (2019).

6. Standard for Emergency and Standby Power Systems (National Fire Protection Association, 2022). *https://www.nfpa.org/codes-and-standards/ all-codes-and-standards/list-of-codes-and-standards/detail?code=110.*

7. Haug, P., Kreitz, B., Koj, M. & Turek, T. Process modelling of an alkaline water electrolyzer. *Int. J. Hydrogen Energy* **42**, 15689–15707 (2017).

8. Brauns, J. & Turek, T. Alkaline water electrolysis powered by renewable energy: A review. *Processes* **8**, (2020).

9. Wong, C. Y. *et al.* Additives in proton exchange membranes for low- and high-temperature fuel cell applications: A review. *Int. J. Hydrogen Energy* **44**, 6116–6135 (2019).

10. Kempf, A. & Martin, W. R. B. Measurement of the thermal properties of TiFe0.85Mn0.15 and its hydrides. *Int. J. Hydrogen Energy* **11**, 107–116 (1986).

11. Mintz, M. H., Vaknin, S., Biderman, S. & Hadari, Z. Hydrides of ternary TiFexM1-x(M=Cr, Mn, Co, Ni) intermetallics. *J. Appl. Phys.* **52**, 463–467 (1981).

12. Dematteis, E. M. *et al.* Fundamental hydrogen storage properties of TiFe-alloy with partial

substitution of Fe by Ti and Mn. *J. Alloys Compd.* **874**, 159925 (2021).

13. Herbrig, K., Röntzsch, L., Pohlmann, C., Weißgärber, T. & Kieback, B. Hydrogen storage systems based on hydride-graphite composites: Computer simulation and experimental validation. *Int. J. Hydrogen Energy* **38**, 7026–7036 (2013).

14. Vanhanen, J. P., Hagström, M. T. & Lund, P. D. Combined hydrogen compressing and heat transforming through metal hydrides. *Int. J. Hydrogen Energy* **24**, 441–448 (1999).

15. Voskuilen, T. G., Waters, E. L. & Pourpoint, T. L. A comprehensive approach for alloy selection in metal hydride thermal systems. *Int. J. Hydrogen Energy* **39**, 13240–13254 (2014).

16. Muthukumar, P., Maiya, M. P. & Murthy, S. S. Experiments on a metal hydride-based hydrogen storage device. *Int. J. Hydrogen Energy* **30**, 1569–1581 (2005).

17. Satheesh, A. & Muthukumar, P. Performance investigation of double-stage metal hydride based heat pump. *Appl. Therm. Eng.* **30**, 2698–2707 (2010).

18. Satheesh, A., Muthukumar, P. & Dewan, A. Computational study of metal hydride cooling system. *Int. J. Hydrogen Energy* **34**, 3164–3172 (2009).

19. Allendorf, M. D., Horton, R., Stavila, V. & Witman, M. Assessment of tank designs for hydrogen storage on heavy duty vehicles using metal hydrides. *Sandia Rep. SAND2023-05851* (2023).

20. Mark D Allendorf *et al.* HyMARC : SNL Activities (ST233). in *2023 Annual Merit Review and Peer Evaluation Meeting* 1–39 (2023).

21. Ulf Bossel, B. E. *Energy and the Hydrogen Economy*. (2003) doi:10.1016/S1464-2859(03)00606-0.

22. Corgnale, C., Hardy, B. J., Tamburello, D. A., Garrison, S. L. & Anton, D. L. Acceptability envelope for metal hydride-based hydrogen storage systems. *Int. J. Hydrogen Energy* **37**, 2812–2824 (2012).

23. Brooks, K. *et al.* PNNL Development and Analysis of Material-Based Hydrogen Storage Systems for the Hydrogen Storage Engineering Center of Excellence. (2016).

24. Klymyshyn, N. A., Brooks, K. & Barrett, N. Methods for Estimating Hydrogen Fuel Tank

Characteristics. *J. Press. Vessel Technol. Trans. ASME* **146**, 1–10 (2024).

25. Brooks, K. P., Sprik, S. J., Tamburello, D. A. & Thornton, M. J. Design tool for estimating metal hydride storage system characteristics for light-duty hydrogen fuel cell vehicles. *Int. J. Hydrogen Energy* **45**, 24917–24927 (2020).

26. Peng, P. *et al.* Cost and potential of metal–organic frameworks for hydrogen back-up power supply. *Nat. Energy* **7**, 448–458 (2022).

27. Woods, D. R. *Rules of Thumb in Engineering Practice*.

28. U.S. Berau of Labor Statistics. *Table B-3. Average hourly and weekly earnings of all employees on private nonfarm payrolls by industry sector, seasonally adjusted. US Census Bureau Report* https://www.bls.gov/news.release/empsit.t19.htm (2024).

29. Geng, Y., Han, X., Zhang, H. & Shi, L. Optimization and cost analysis of thickness of vacuum insulation panel for structural insulating panel buildings in cold climates. *J. Build. Eng.* **33**, 101853 (2021).

30. Lakatos, Á. & Kovács, Z. Comparison of thermal insulation performance of vacuum insulation panels with EPS protection layers measured with different methods. *Energy Build.* **236**, (2021).

31. Wernery, J., Mancebo, F., Malfait, W. J., O'Connor, M. & Jelle, B. P. The economics of thermal superinsulation in buildings. *Energy Build.* **253**, 111506 (2021).

32. Hua, T. Q. *et al.* Technical assessment of compressed hydrogen storage tank systems for automotive applications. *Int. J. Hydrogen Energy* **36**, 3037–3049 (2011).

33. 2023 Commercial Industrial Land Values. 2–3 https://cms6.revize.com/revize/breitungtwp/C I 2023 Binder2.pdf (2023).

34. Chemical Engineering Plant Cost Index. https://personalpages.manchester.ac.uk/staff/tom.rodgers/Interactive_graphs/CEPCI.html?reactors/CEPCI/index.html.

35. Shaner, M. R., Atwater, H. A., Lewis, N. S. & McFarland, E. W. A comparative technoeconomic analysis of renewable hydrogen production using solar energy. *Energy Environ. Sci.* **9**, 2354–2371 (2016).

36. Spurgeon, J. M. & Kumar, B. A comparative technoeconomic analysis of pathways for commercial electrochemical CO2 reduction to liquid products. *Energy Environ. Sci.* **11**, 1536–1551 (2018).

37. Parks, G., Boyd, R., Cornish, J. & Remick, R. *Hydrogen Station Compression, Storage, and Dispensing Technical Status and Costs. Related Information: Independent review published for the U.S. Department of Energy Hydrogen and Fuel Cells Program* http://www.osti.gov/scitech//servlets/purl/1130621/ (2014).

38. LaChance, J., Houf, W., Middleton, B. & Fluer, L. Analyses to support development of risk-informed separation distances for hydrogen codes and standards. *Sand2009-0874* 130 (2009).

39. Proceedings of the DOE chemical/hydrogen energy contractor review systems. (1978).

40. Goodell, P. D., Sandrock, G. D. & Huston, E. L. Kinetic and Dynamic Aspects of Rechargeable Metal Hydrides. *J. less-common Met.* **73**, 135–142 (1980).

41. Reilly, J. J., Johnson, J. R., Lynch, J. F. & Reidinger, F. Irreversible effects in the FeTi-H system. *J. Less-Common Met.* **89**, 505–512 (1983).

42. Ahn, H. J., Lee, S. M. & J., L. Intrinsic degradation of FeTi by thermally induced hydrogen absorption - desorption cycling. *J. Less-Common Met.* **142**, 253–261 (1988).

43. Johnson, J. R. & Reilly, J. J. Use of Manganese Substituted Ferrotitanium Alloys for Energy Storage. *Proc. Int. Conf. Altern. Energy Sources* **v**, 3739–3769 (1977).

44. Dehouche, Z. *et al.* Influence of cycling on the thermodynamic and structure properties of nanocrystalline magnesium based hydride. *J. Alloys Compd.* **305**, 264–271 (2000).

45. Reiser, A., Bogdanović, B. & Schlichte, K. Application of Mg-based metal-hydrides as heat energy storage systems. *Int. J. Hydrogen Energy* **25**, 425–430 (2000).

46. Dehouche, Z., Djaozandry, R., Goyette, J. & Bose, T. K. Thermal cyclic charge and discharge stability of nanocrystalline Mg2Ni alloy. *J. Alloys Compd.* **288**, 312–318 (1999).

47. Friedlmeier, G., Manthey, A., Wanner, M. & Groll, M. Cyclic stability of various application-relevant metal hydrides. *J. Alloys Compd.* **231**, 880–887 (1995).

48. Cheng, H. H. *et al.* Effect of hydrogen absorption/desorption cycling on hydrogen storage performance of LaNi4.25Al0.75. *J. Alloys Compd.* **453**, 448–452 (2008).

49. Kim, K. J., Montoya, B., Razani, A. & Lee, K. H. Metal hydride compacts of improved thermal conductivity. *Int. J. Hydrogen Energy* **26**, 609–613 (2001).

50. Tarasov, B. P. *et al.* Metal hydride hydrogen storage and compression systems for energy storage technologies. *Int. J. Hydrogen Energy* **46**, 13647–13657 (2021).

51. Chi, J. & Yu, H. Water electrolysis based on renewable energy for hydrogen production. *Cuihua Xuebao/Chinese J. Catal.* **39**, 390–394 (2018).

52. Shiva Kumar, S. & Himabindu, V. Hydrogen production by PEM water electrolysis – A review. *Mater. Sci. Energy Technol.* **2**, 442–454 (2019).

53. Esposito, D. V. Membraneless Electrolyzers for Low-Cost Hydrogen Production in a Renewable Energy Future. *Joule* **1**, 651–658 (2017).

54. Yates, J. *et al.* Techno-economic Analysis of Hydrogen Electrolysis from Off-Grid Stand-Alone Photovoltaics Incorporating Uncertainty Analysis. *Cell Reports Phys. Sci.* **1**, 100209 (2020).

55. Andersson, J. & Grönkvist, S. Large-scale storage of hydrogen. *Int. J. Hydrogen Energy* **44**, 11901–11919 (2019).
